# Stereoselective Syntheses and Application of Chiral Bi- and Tridentate Ligands Derived from (+)-Sabinol

**DOI:** 10.3390/molecules23040771

**Published:** 2018-03-27

**Authors:** Yerbolat Tashenov, Mathias Daniels, Koen Robeyns, Luc Van Meervelt, Wim Dehaen, Yerlan M. Suleimen, Zsolt Szakonyi

**Affiliations:** 1 Institute of Applied Chemistry, Chemistry Department of L.N. Gumilyov Eurasian National University, Munaitpassov st., 5, 010008 Astana, The Republic of Kazakhstan; tashenov_yeo@edu.enu.kz (Y.T.); Suleimen_em@enu.kz (Y.M.S.); 2 KU Leuven, Department of Chemistry, Celestijnenlaan 200F, B-3001 Leuven, Belgium; mathias.daniels@kuleuven.be (M.D.); luc.vanmeervelt@kuleuven.be (L.V.M.); wim.dehaen@kuleuven.be (W.D.); 3 IMCN, Molecules Solids and Reactivity division (MOST), Université catholique de Louvain, Place Pasteur 1, B-1348 Louvain-la-Neuve, Belgium; koen.robeyns@uclouvain.be; 4 Institute of Pharmaceutical Chemistry, University of Szeged, Eötvös u. 6, H-6720 Szeged, Hungary; 5 Interdisciplinary Centre of Natural Products, University of Szeged, H 6720 Szeged, Hungary

**Keywords:** sabinol, terpenoid, catalyst, chiral ligand, triol, aminodiol, asymmetric catalysis

## Abstract

A library of bidentate diols, as well as tridentate triols and aminodiols, derived from (+)-sabinol, was synthesized in a stereoselective manner. Sabinol was transformed into allylic trichloroacetamide via Overman rearrangement of the corresponding trichloroacetimidate. After changing the protecting group to Boc, the enamine was subjected to stereospecific dihydroxylation with OsO_4_/NMO, resulting in the (1*R*,2*R*,3*R*,5*R*)-aminodiol diastereomer. The obtained primary aminodiol was transformed to a secondary analogue. The ring closure of the *N*-benzyl-substituted aminodiol with formaldehyde was investigated and regioselective formation of the spiro-oxazolidine ring was observed. Hydroboration or dihydroxylation of sabinol or its benzyl ether with OsO_4_/NMO resulted in the formation of sabinane-based diols and triols following a highly stereospecific reaction. Treatment of sabinol with *m*-CPBA afforded *O*-benzoyl triol as a diastereoisomer of the directly dihydroxylated product, instead of the expected epoxy alcohol. The resulting aminodiols, diol, and triols were applied as chiral catalysts in the reaction of diethylzinc and benzaldehyde from moderate to good selectivity.

## 1. Introduction

In recent years, the discovery and application of new chiral auxiliaries and catalysts have become a crucial question in stereoselective syntheses [1,2,3,4]. To achieve new, efficient, and commercially available chiral catalysts, natural chiral terpenes [5], including (+)-pulegone [6,7,8], α- and β-pinene [9,10,11,12,13,14], and fenchone-camphor [15,16,17,18,19] have proved to be excellent sources. Starting from these readily available natural sources, several powerful chiral catalysts, including various di- and trifunctional synthons, such as 1,3-aminoalcohols and aminodiols, have been applied in stereoselective syntheses [5,20]. 

Besides their importance in enantioselective catalysis, 3-amino-1,2-diols are good starting materials for the synthesis of various heterocyclic ring systems, such as 1,3-oxazines or oxazolidines [8,11,21,22,23,24,25]. Several mono- or bicyclic aminodiol derivatives possess remarkable biological activity. The Abbott aminodiol was also found to be a useful building block for the synthesis of the potent renin inhibitor Zankiren [26]. Some 3-amino-1,2-diols have been investigated as selective antagonists on receptor P2X_1_ [27]. Cytoxazone is a naturally occurring heterocyclic aminodiol derivative isolated from *Streptomyces* species [28,29], and expresses cytokine modulator activity by inhibiting the signaling pathway of Th2 cells [30,31]. Some bicyclic aminodiol-based carbocyclic nucleoside analogues exert antiviral activity [32].

Monoterpene-based diols or triols have also proved to be easily available, good chiral auxiliaries and catalysts [33,34]. Some of the terpenoid diols also possess marked biological, e.g., antimicrobial, antifungal or enzyme inhibitor activities [35,36,37].

Similar to pinane- and carane-based allylic alcohols, (+)-sabinol and its acetate are available from the essential oil of several plants e.g., *Juniperus sabina* L. in a large scale [38,39,40]. Although this interesting monoterpene derivative has been intensively studied from the biological point of view, it was scarcely investigated for its chemical transformations [41].

Our present aim was to synthesize a library of sabinane-based chiral di- and trifunctional synthons, such as 3-amino-1,2-diols, diols, and triols starting from (+)-sabinol, achieved from a natural source. We also decided to evaluate the resulting synthons as catalysts in the asymmetric addition of Et_2_Zn to benzaldehyde. The planned aminodiols, diols, and triols may serve as useful building blocks for the synthesis of new heterocyclic ring systems and biologically active compounds.

## 2. Results

### 2.1. Synthesis and Transformations of Sabinol-Based 3-Amino-1,2-Diols

(+)-Sabinol **1**, the key starting material, was isolated from the essential oil of *Juniperus sabina* L. according to the literature procedure, and its purity was found to be >98% based on GC analysis [38,39,40].

**Scheme 1:**
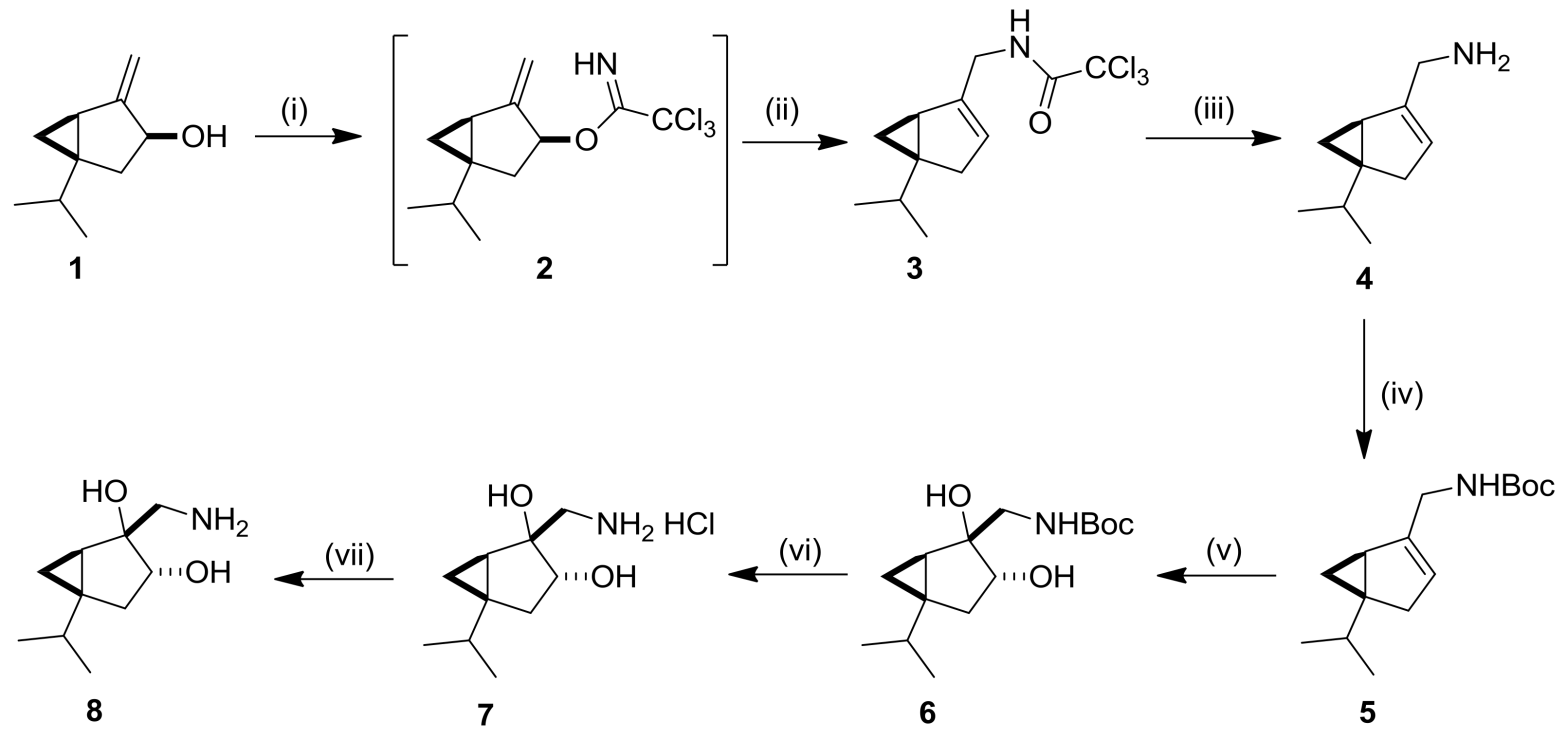
*Reagents and conditions*: (i) CCl_3_CN, DBU, dry CH_2_Cl_2_, rt, 2 h; (ii) anhydrous K_2_CO_3_, dry xylene, reflux, 12 h, 77%; (iii) 2 N aq NaOH, EtOH, rt, 2 h, 72%; (iv) Boc_2_O, TEA, DMAP, THF, rt, 22 h, 95%; (v) OsO_4_, NMO, acetone/H_2_O, rt, 12 h, 93%; (vi) 5% aq HCl, Et_2_O, rt, 24 h, 77%; (vii) 15% aq KOH, CH_2_Cl_2_, rt, 83%.

Sabinol was transformed into trichloroacetimidate **2** in the presence of trichloroacetonitrile and DBU as a strong base (Scheme 1) [8,11]. **2** underwent Overman rearrangement by heating in the presence of K_2_CO_3_, resulting in protected allylamine **3** [42]. Since we have found difficulties during the deprotection of *N*-trichloroacetyl aminodiols in our recent studies [8], we have changed the protecting group to Boc in a two-step process via enamine **4**. Stereospecific dihydroxylation of **5**, applying OsO_4_ as the catalyst and NMO as the oxidant, produced protected (1*R*,2*R*,3*R*,5*R*)-aminodiol **6** as a single diastereomer (based on the ^1^H NMR study of the crude product). Acid-catalyzed removal of the protecting group resulted in primary aminodiol hydrochloride **7** with 41% overall yield.

**Figure 1:**
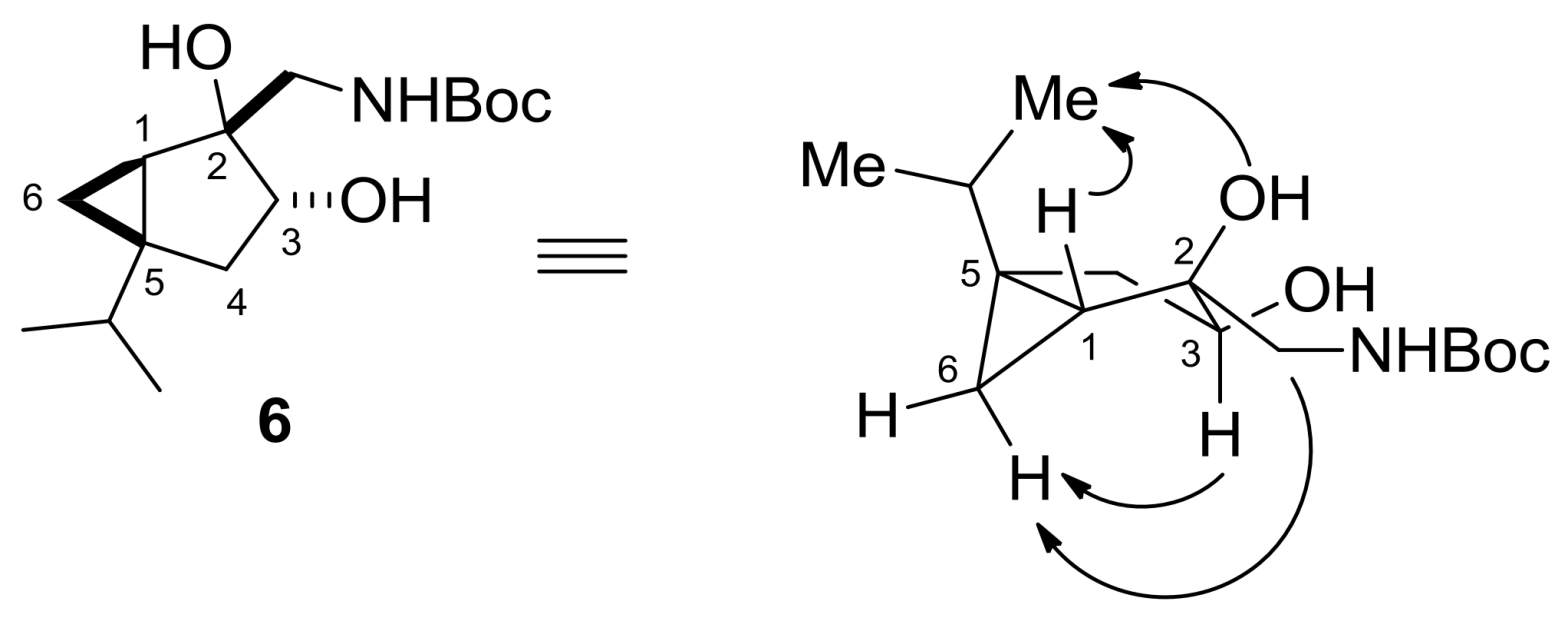
Determination of the relative configuration of **6** by NOESY.

The relative configuration of **6** was determined by NOESY spectral analysis. Clear NOE correlations were observed between Me of the isopropyl group at position 5 and H-1, OH-2, as well as between H-6 and H-3, *CH_2_*NHBoc. Therefore, the structure of **6** was assigned as shown in Figure 1.

**Scheme 2:**
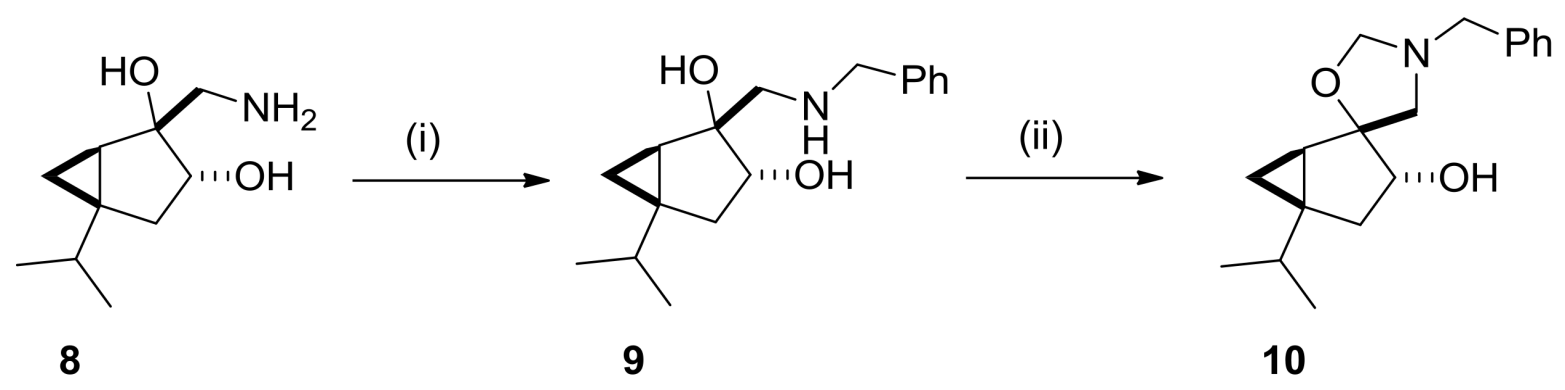
*Reagents and conditions*: (i) PhCHO, dry EtOH, 2 h, then NaBH_4_, EtOH, 48 h, rt, 56%; (ii) 35% aq CH_2_O, Et_2_O, rt, 1 h, 90%.

Aminodiol **8** was transformed to *N*-benzyl derivative **9** by reductive alkylation via condensation with benzaldehyde, followed by subsequent reduction with sodium borohydride (Scheme 2). Next, the regioselectivity of the ring closure of **9** with formaldehyde was investigated. The reaction has proved to be highly regioselective, resulting only in spiro-oxazolidine **10**, while the fused 1,3-oxazine structure could not be detected from the crude product by means of ^1^H NMR spectroscopy. This observation shows similarity with earlier results we have obtained after ring closure of pinane-based 3-amino-1,2-diols [23], and it is in contrast with those of carane-based analogues [24,25].

### 2.2. Synthesis and Transformations of Sabinol-Based Diols and Triols

Since monoterpenic diols and triols might serve as chiral catalysts or could be used as excellent starting materials for the synthesis of more complex 1,3-heterocycles [34,43], we decided to explore the preparation and some transformations of sabinane-based diols and triols starting from (+)-sabinol **1**.

**Scheme 3:**
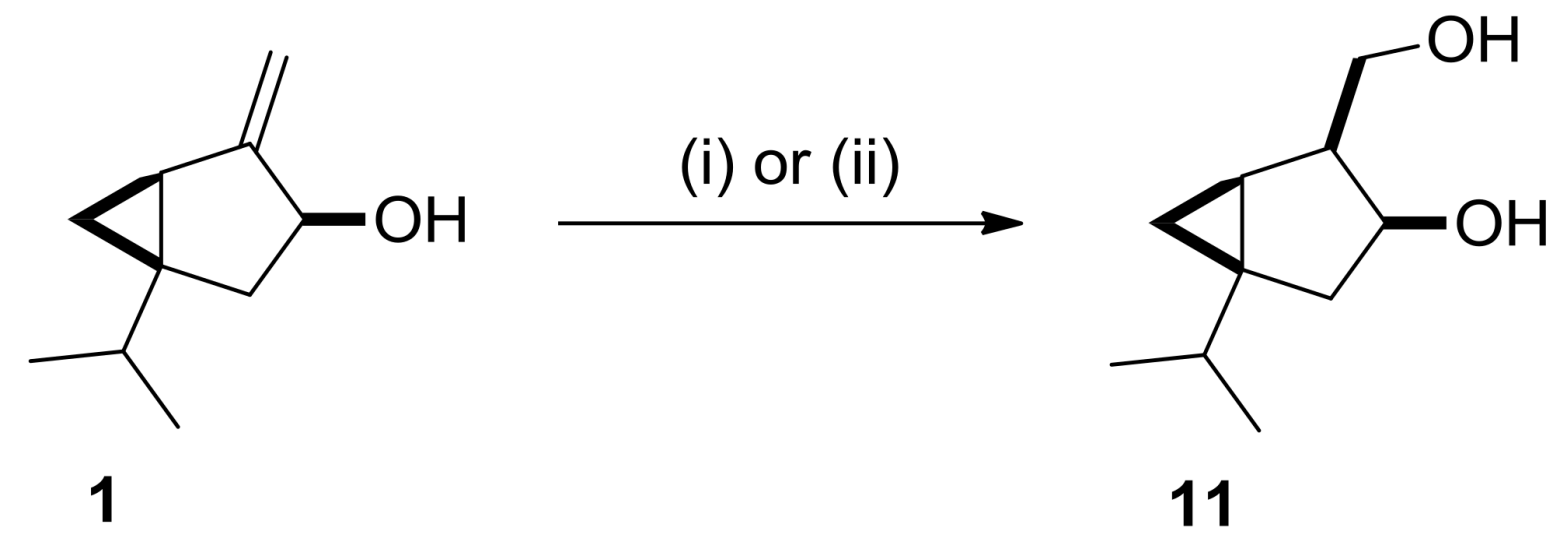
(i) BH_3_∙THF, THF, 0 °C to rt, 2.5 h, then NaOH/H_2_O_2_/H_2_O, THF, rt, 30 min, 47%; (ii) BH_3_·Me_2_S, THF, 0 °C to rt, 18 h, then NaOH/H_2_O_2_/H_2_O, THF, rt, 30 min, 70%.

**Figure 2:**
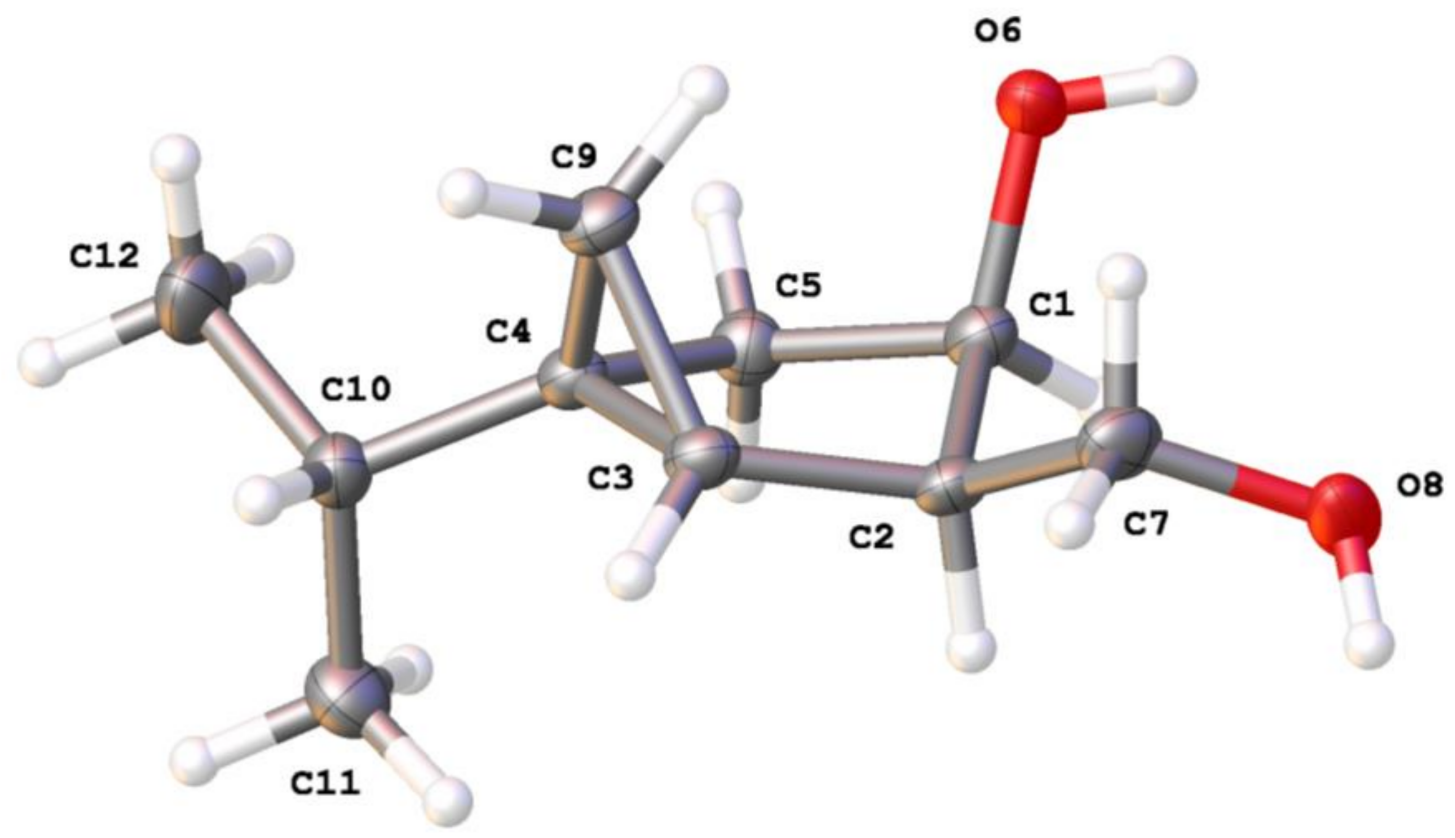
Crystal structure of compound **11**. Thermal ellipsoids are drawn at the 50% probability level.

Hydroboration reaction of **1** with BH_3_∙THF or BH_3_∙Me_2_S followed by treatment with H_2_O_2_ resulted in *cis* bicyclic diol **11** in a highly stereospecific reaction (Scheme 3). Beside the NOESY spectral structural analysis, the relative configuration of **11** was determined by X-ray crystallography (Figure 2).

The planes of the two rings in **11** make an angle of 76.90(14)°. The cyclopentane ring has an envelope conformation with atom C1 as the tip. The substituent O6–H6 occupies an axial position on the envelope tip, whereas the isopropyl and O8–H8 substituents are in equatorial positions. In the crystal packing, both hydroxyl groups are involved in hydrogen bonds [O6…O8(−1/2 + *x*, −1/2 − *y*, 1 − *z*) = 2.6796(18) Å, O8…O6(1 + *x*, *y*, *z*) = 2.7223(16) Å], resulting in a double chain of molecules running in the *a*-axis direction (Figure S1).

**Scheme 4:**
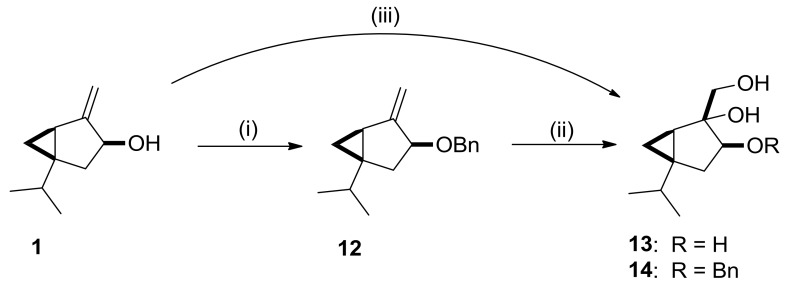
(i) BnBr, NaH, THF, reflux, 1 h, 92%; (ii) OsO_4_, NMO, acetone/H_2_O, rt, 12 h, 44%; (ii) OsO_4_, NMO, acetone/H_2_O, rt, 12 h, 81%.

The synthesis of sabinane-based triols also started from **1**. (+)-Sabinol was treated with the OsO_4_/NMO system providing triol **13** in a highly stereospecific reaction. In order to compare the catalytic importance of hydroxyl functions, *O*-benzyl derivative **14** was also prepared via benzylation of **1** with benzyl bromide, followed by stereospecific dihydroxylation (Scheme 4).

**Scheme 5:**
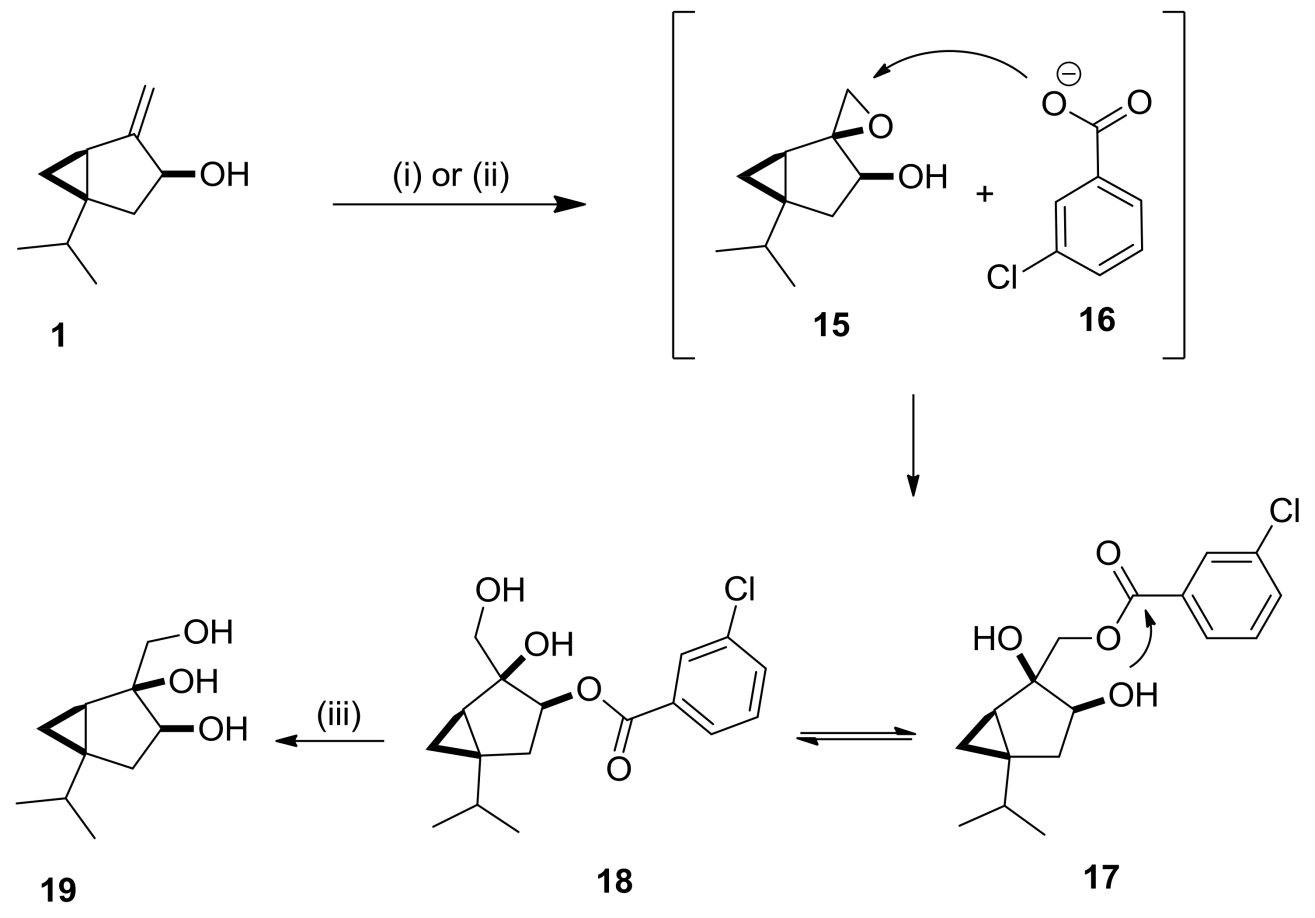
(i) *m*-CPBA, CH_2_Cl_2_, Na_2_HPO_4_ buffer sol., (pH = 9.2), rt, 1 h, work up with 10% NaOH/H_2_O, 37% (mixture of **17** and **18**); (ii) *m*-CPBA, CH_2_Cl_2_, Na_2_HPO_4_. buffer sol. (pH = 9.2), rt, 1 h, work up with sat. NaHCO_3_/H_2_O, 38% (**18**); (iii) 10% NaOH/H_2_O, EtOH, rt, 30 min, 91%.

Treatment of sabinol **1** with *m*-CPBA did not yield the desired epoxide **15**. Rather, the generated epoxide underwent an in situ ring-opening process after the electrophilic attack of the formed *m*-chlorobenzoic acid, resulting in ester **17** (Scheme 5) [44,45,46]. The intermediate epoxide could not be isolated even after applying miscellaneous conditions, such as varying temperature, NaHCO_3_ or Na_2_CO_3_, or buffer solutions. **17** easily underwent acyl migration under the applied conditions, affording its structural isomer **18**. Interesting to note that while under slightly alkaline work up conditions (sat. NaHCO_3_ solution) only isomer **18** was isolated, applying strong alkaline conditions (10% NaOH solution), the mixture of two regioisomers was isolated. Acyl migration of the pure products could also be observed during ^13^C NMR analysis in CDCl_3_ solution. It must be mentioned that Garside et al. reported a similar reaction of sabinol with peracetic acid; however, they observed ring rearrangement and formation of acetyl *p*-menthane-1,2,4-triol [47].

The regioisomeric relation of **17** and **18** was determined by their hydrolysis under alkaline conditions, which furnished stereohomogenic compound **19**, whereas the relative configuration of **19** was assigned by NOESY spectral structural analysis and **19** was found to be a diastereoisomer of triol **13**.

### 2.3. Application of Sabinol-Based Aminodiol, Diol and Triol Derivatives as Chiral Ligands in the Enantioselective Addition of Diethylzinc to Benzaldehyde

**Scheme 6:**
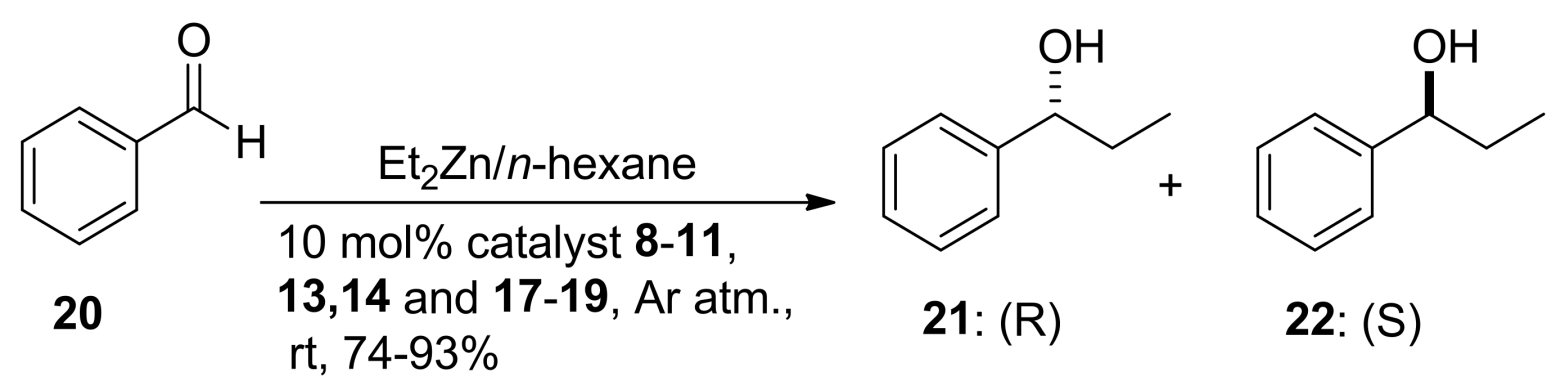
Catalyzed addition of diethylzinc to benzaldehyde.

Application of the prepared 3-amino-1,2-diols (**8**–**10**), 1,3-diol (**11**), and triols (**13**,**14** and **17**–**19**) as catalysts in the ethylation of benzaldehyde resulted in 1-phenyl-1-propanol enantiomers **21** and **22** (Scheme 6).

The enantiomeric purity of the secondary alcohol obtained was determined by HPLC analysis [7]. Catalysts were applied in 10% molar ratio and reactions were carried out in *n*-hexane at room temperature. The results are presented in Table 1.

In the addition of Et_2_Zn in *n*-hexane solution to benzaldehyde, diol **11**, or triols **13**, **14**, and **17**–**19** afforded the corresponding alcohols with low enantioselectivities. The stereoselectivity could not be improved either by changing the solvent to toluene or lowering the reaction temperature. Increased, but still moderate asymmetric induction was observed when aminodiol derivatives **8**–**10** were used. The formation of *S* enantiomer **22** was predominant in all cases.

## 3. Experimental Section

### 3.1. Materials and Methods

^1^H and ^13^C NMR spectra were recorded on a Bruker Avance DRX 300 and 500 spectrometer [δ = 0 (TMS)] (Bruker Corp., Billerica, MA, USA) in the solvents indicated. Chemical shifts are expressed in ppm (δ) relative to TMS as internal reference. *J* values are given in Hz. Elemental analyses were performed on a Perkin-Elmer 2400 Elemental Analyzer (PerkinElmer Inc., Waltham, MA, USA). Chiral HPLC analysis was performed without derivatization by JASCO LC-4000 system on a Chiralcel OD-H column (250 × 4.6 mm, Jasco Europe S.R.L., Cremella, Italy). UV detection was monitored at 215 nm. Optical rotations were obtained with a Perkin-Elmer 341 polarimeter (PerkinElmer Inc., Shelton, CT, USA). Melting points were determined on a Kofler apparatus (Nagema, Dresden, Germany) and are uncorrected. Chromatographic separations were carried out on Merck Kieselgel 60 (230–400 mesh ASTM, Merck Ltd., Budapest, Hungary). Reactions were monitored with Merck Kieselgel 60 F254-precoated TLC plates (0.25 mm thickness). All chemicals and solvents were used as supplied.

Sabinol **1** was isolated from the essential oil of *Juniperus sabina* L. according to a literature process [38,39,40].

*Synthesis of 2,2,2-trichloro-N-(((1R,5S)-5-isopropylbicyclo[3.1.0]hex-2-en-2-yl)methyl)acetamide* (**3**). To a solution of sabinol **1** (4.00 g, 26.27 mmoL) in dry CH_2_Cl_2_ (50 mL) 1,8-diazabicycloundec-7-ene (2 mL, 13.39 mmoL) and CCl_3_CN (4.68 mL, 46.67 mmoL) were added at 0 °C. The reaction mixture was then allowed to warm to room temperature and stirred for 2 h. When the reaction was completed (monitored by means of TLC), the mixture was concentrated to a volume of 20 mL and then filtered through a short pad of silica gel, washing with CH_2_Cl_2_. Evaporation of the solvent in vacuum gave a brown oil, which was immediately dissolved in dry xylene (200 mL). To this solution, anhydrous K_2_CO_3_ (1.00 g, 7.23 mmoL) was added, and the mixture was treated at reflux temperature overnight. The obtained solution was then filtered through a Celite pad, washed, and concentrated under reduced pressure. The obtained crude product was purified by column chromatography on silica gel (R_f_ = 0.52; n-hexane/EtOAc = 9/1).

Compound **3**: 6.03 g (77%); pale-yellow crystalline powder; mp: 45–47 °C; ${\left[\alpha \right]}_{D}^{20}$ = +7 (*c* = 0.250; MeOH). ^1^H NMR (300 MHz, CDCl_3_) δ (ppm): 0.08 (1H, t, *J* = 3.4 Hz), 0.84 (1H, dd, *J* = 3.8, 7.5 Hz), 0.90 (3H, d, *J* = 6.9 Hz), 0.95 (3H, d, *J* = 6.8 Hz), 1.39–1.52 (2H, m), 2.25 (1H, d, *J* = 17.7 Hz), 2.45 (1H, dd, *J* = 2.2, 17.7 Hz), 3.98–4.16 (2H, m), 5.26 (1H, s), 6.79 (1H, *br* s). ^13^C NMR (75 MHz, CDCl_3_) δ (ppm): 19.9, 20.2, 21.8, 28.4, 32.8, 34.0, 36.4, 41.8, 92.9, 124.0, 144.1, 161.8. Anal. Calcd for C_12_H_16_Cl_3_NO: C 48.59; H 5.44; Cl 35.86; N 4.72. Found: C 48.65; H 5.52; Cl 35.63; N 4.68.

*Synthesis of ((1R,5S)-5-isopropylbicyclo[3.1.0]hex-2-en-2-yl)methanamine* (**4**). A solution of acetamide **3** (5.95 g, 20 mmoL) in ethanol (30 mL) was stirred with 2 N aqueous NaOH solution (30 mL) at room temperature for 2 h. The reaction mixture was then concentrated to an aqueous residue which was extracted with CH_2_Cl_2_ (3 × 30 mL). The organic layer was dried (Na_2_SO_4_) and evaporated to dryness. Compound **4** was used without further purification.

Compound **4**: 2.18 g (72%); yellow oil; ${\left[\alpha \right]}_{D}^{20}$ = −5 (*c* = 0.250; MeOH). ^1^H NMR (500 MHz, CDCl_3_) δ (ppm): 0.05 (1H, t, *J* = 3.2 Hz), 0.80 (1H, dd, *J* = 3.5, 7.5 Hz), 0.90 (3H, d, *J* = 6.8 Hz), 0.95 (3H, d, *J* = 6.8 Hz), 1.41–1.47 (4H, m), 2.22 (1H, d, *J* = 17.3 Hz), 2.41 (1H, dd, *J* = 2.0, 17.3 Hz), 3.37 (2H, dd, *J* = 15.4, 15.6 Hz), 5.13 (1H, s). ^13^C NMR (125.8 MHz, CDCl_3_) δ (ppm): 19.8, 20.1, 21.7, 28.7, 32.8, 33.7, 36.4, 42.7, 120.0, 151.2. Anal. Calcd for C_10_H_17_N: C 79.41; H 11.33; N 9.26. Found: C 79.63; H 11.45; N 9.01.

*Synthesis of tert-butyl (((1R,5S)-5-isopropylbicyclo[3.1.0]hex-2-en-2-yl)methyl)carbamate* (**5**). To a solution of **4** (2.00 g, 13.22 mmoL) TEA (3.34 g, 33.05 mmoL), DMAP (0.16 g, 1.32 mmoL) in THF (100 mL) and Boc_2_O (3.17 g, 14.54 mmoL) were added at room temperature. The reaction mixture was stirred for 22 h and then concentrated under reduced pressure. The crude product was purified by column chromatography on silica gel (R_f_ = 0.50; n-hexane/EtOAc = 9/1).

Compound **5**: 3.16 g (95%); pale-yellow oil; ${\left[\alpha \right]}_{D}^{20}$ = +6 (*c* = 0.250; MeOH). ^1^H NMR (500 MHz, CDCl_3_) δ (ppm): 0.04 (1H, t, *J* = 3.1 Hz), 0.80 (1H, dd, *J* = 3.7, 7.5 Hz), 0.89 (3H, d, *J* = 6.8 Hz), 0.94 (3H, d, *J* = 6.7 Hz), 1.44–1.47 (2H, m), 1.45 (9H, s), 2.21 (1H, d, *J* = 17.5 Hz), 2.40 (1H, dd, *J* = 2.0, 17.4 Hz), 3.81 (2H, s), 4.59 (1H, *br* s), 5.15 (1H, s). ^13^C NMR (125.8 MHz, CDCl_3_) δ (ppm): 19.8, 20.1, 21.6, 28.4, 28.4, 32.7, 33.7, 36.3, 41.1, 79.2, 122.1, 146.3, 155.9. Anal. Calcd for C_15_H_25_NO_2_: C 71.67; H 10.02; N 5.57. Found: C 71.88; H 10.20; N 5.41.

*Synthesis of tert-butyl (((1R,2R,3R,5R)-2,3-dihydroxy-5-isopropylbicyclo[3.1.0]hexan-2-yl)methyl)carbamate* (**6**). To **5** (3.00 g, 11.93 mmoL) in acetone (50 mL) 4-methylmorpholine N-oxide (10 mL, 50% aqueous solution) and OsO_4_ (4 mL, 2% tert-BuOH solution) were added. The reaction mixture was stirred for 12 h at room temperature. The reaction was then quenched by the addition of saturated aqueous Na_2_SO_3_ (50 mL) and extracted with EtOAc (3 × 50 mL). The combined organic phase was dried (Na_2_SO_4_) and evaporated, then the crude residue was purified by column chromatography (R_f_ = 0.43; n-hexane/EtOAc = 1:1).

Compound **6**: 3.16 g (93%); off-white crystalline powder; mp: 68–70 °C; ${\left[\alpha \right]}_{D}^{20}$ = +38 (*c* = 0.250; MeOH). ^1^H NMR (500 MHz, CDCl_3_) δ (ppm): 0.30 (1H, dd, *J* = 3.9, 5.4 Hz), 0.38–0.44 (1H, m), 0.88 (3H, d, *J* = 6.9 Hz), 0.97 (3H, d, *J* = 6.8 Hz), 1.19 (1H, dd, *J* = 3.6, 8.5 Hz), 1.34 (1H, quint, *J* = 6.8 Hz), 1.45 (9H, s), 1.66 (1H, dd, *J* = 9.6, 11.5 Hz), 2.05 (1H, dd, *J* = 7.3, 12.2 Hz), 3.22 (1H, dd, *J* = 6.8, 14.2 Hz), 3.30 (1H, dd, *J* = 5.2, 14.3 Hz), 3.58 (1H, t, *J* = 8.1 Hz), 5.03 (1H, *br* s). ^13^C NMR (125.8 MHz, CDCl_3_) δ (ppm): 13.3, 19.7, 19.8, 28.4, 28.9, 31.3, 32.6, 34.4, 47.0, 73.3, 79.1, 80.0. Anal. calcd for C_15_H_27_NO_4_: C 63.13; H 9.54; N 4.91. Found: C 63.29; H 9.65; N 4.73.

*Synthesis of (1R,2R,3R,5R)-2-aminomethyl-5-isopropylbicyclo[3.1.0]hexane-2,3-diol hydrochloride* (**7**). A solution of **6** (1.20 g, 4.20 mmoL) in Et_2_O (30 mL) was stirred with 30 mL of 5% aqueous HCl at room temperature. After 24 h, the two phases were separated, the aqueous phase was washed with Et_2_O (3 × 30 mL) and then evaporated to dryness. Crystals formed were thoroughly washed with Et_2_O.

Compound **7**: 0.72 g (77%); colorless crystals; mp: 146–148 °C; ${\left[\alpha \right]}_{D}^{20}$ = +42 (*c* = 0.250; MeOH). ^1^H NMR (500 MHz, D_2_O) δ (ppm): 0.41 (1H, dd, *J* = 3.9, 5.6 Hz), 0.54–0.59 (1H, m), 0.85 (3H, d, *J* = 6.9 Hz), 0.97 (3H, d, *J* = 6.8 Hz), 1.30 (1H, dd, *J* = 3.6, 8.5 Hz), 1.47 (1H, septet, *J* = 6.8 Hz), 1.75 (1H, dd, *J* = 10.0, 11.71 Hz), 2.15 (1H, dd, *J* = 7.4, 12.3 Hz), 3.10 (2H, s), 3.66 (1H, dd, *J* = 7.9, 8.7 Hz). ^13^C NMR (125.8 MHz, D_2_O) δ (ppm): 11.8, 18.6, 19.1, 26.7, 31.6, 32.0, 33.2, 45.0, 72.8, 77.0. Anal. calcd for C_10_H_20_ClNO_2_: C 54.17; H 9.09; N 6.32. Found: C 54.28; H 9.29; N 6.01.

*Synthesis of (1R,2R,3R,5R)-2-((benzylamino)methyl)-5-isopropylbicyclo[3.1.0]hexane-2,3-diol* (**9**). To a solution of aminodiol **8** liberated from **7** (0.37 g, 2 mmoL) in dry ethanol (20 mL) benzaldehyde (0.32 g, 3 mmoL) was added in one portion, and the solution was stirred at room temperature for 1 h and then evaporated to dryness. The residual product was dissolved again in dry ethanol (20 mL) and stirred for a further 1 h. Next NaBH_4_ (0.23 g, 6 mmoL) was added in small portions to the mixture under ice cooling. After stirring for 48 h, the mixture was evaporated to dryness and the residue was dissolved in H_2_O and extracted with CH_2_Cl_2_ (3 × 30 mL). The combined organic layer was dried (Na_2_SO_4_), filtered and evaporated to dryness. The obtained crude product was purified by column chromatography on silica gel (R_f_ = 0.21; toluene/ethanol = 4/1).

Compound **9**: 0.31 g (56%); colorless crystals; mp: 111–113 °C; ${\left[\alpha \right]}_{D}^{20}$ = +41 (*c* = 0.250; MeOH). ^1^H NMR (500 MHz, CDCl_3_) δ (ppm): 0.22 (1H, dd, *J* = 4.0, 5.3 Hz), 0.32–0.36 (1H, m), 0.87 (3H, d, *J* = 6.9 Hz), 0.97 (3H, d, *J* = 6.8 Hz), 1.14 (1H, dd, *J* = 3.7, 8.5 Hz), 1.32 (1H, septet, *J* = 6.8 Hz), 1.63 (1H, dd, *J* = 9.8, 11.5 Hz), 2.02 (1H, dd, *J* = 7.3, 12.3 Hz), 2.62 (1H, d, *J* = 11.9 Hz), 2.91 (1H, d, *J* = 11.9 Hz), 3.52 (1H, t, *J* = 7.8 Hz), 3.79 (1H, d, *J* = 13.2 Hz), 3.85 (1H, d, *J* = 13.2 Hz), 7.26–7.36 (5H, m). ^13^C NMR (125.8 MHz, CDCl_3_) δ (ppm): 13.1, 19.6, 19.7, 29.5, 31.7, 32.8, 34.1, 54.2, 55.0, 75.0, 77.6, 127.2, 128.2, 128.5, 139.7. Anal. calcd for C_17_H_25_NO_2_: C 74.14; H 9.15; N 5.09. Found: C 74.25; H 9.10; N 5.15.

*Synthesis of (1R,2R,3R,5R)-3′-benzyl-5-isopropylspiro[bicyclo[3.1.0]hexane-2,5′-oxazolidin]-3-ol* (**10**). To the solution of **9** (0.15 g, 0.54 mmoL) in Et_2_O (5 mL) 35% aqueous formaldehyde solution (2 mL) was added. The reaction mixture was stirred for 1 h at room temperature, followed by making it alkaline with 10% cold aqueous KOH solution and extracted with Et_2_O (3 × 20 mL). The organic layers were combined, washed with saturated NaCl solution (3 × 20 mL) then dried (Na_2_SO_4_), filtered, and evaporated to dryness. The crude product was purified by column chromatography on silica gel (R_f_ = 0.64; toluene/ethanol = 4/1).

Compound **10**: 0.14 g (90%); pale-yellow oil; ${\left[\alpha \right]}_{D}^{20}$ = +63 (*c* = 0.250; MeOH). ^1^H NMR (500 MHz, (CD_3_)_2_SO) δ (ppm): 0.27–0.32 (2H, m), 0.84 (3H, d, *J* = 6.8 Hz), 0.93 (3H, d, *J* = 6.8 Hz), 1.17–1.21 (1H, m), 1.24–1.32 (1H, m), 1.56 (1H, dd, *J* = 10.1, 11.2 Hz), 1.78 (1H, dd, *J* = 7.1, 11.7 Hz), 2.68 (1H, d, *J* = 11.0 Hz), 2.93 (1H, d, *J* = 11.0 Hz), 3.41 (1H, dd, *J* = 9.4, 16.8 Hz), 3.75 (2H, s), 4.13 (1H, d, *J* = 4.5 Hz), 4.25 (1H, d, *J* = 9.5 Hz), 4.41 (1H, d, *J* = 4.4 Hz), 7.22–7.37 (5H, m). ^13^C NMR (125.8 MHz, (CD_3_)_2_SO) δ (ppm): 13.5, 20.2, 20.2, 30.5, 31.9, 32.9, 34.2, 57.9, 58.0, 74.2, 86.7, 87.3, 127.4, 128.7, 129.0, 139.8. Anal. calcd for C_18_H_25_NO_2_: C 75.22; H 8.77; N 4.87. Found: C 75.31; H 8.91; N 4.70.

*Synthesis of (1R,3S,4S,5S)-4-hydroxymethyl-1-isopropylbicyclo[3.1.0]hexan-3-ol* (**11**). Method A: to a cooled (0 °C) solution of sabinol 1 (0.15 g, 1 mmoL) in 3 mL dry THF under nitrogen atmosphere, a solution of 1 M borane THF complex (2 mL) was injected dropwise. After completing the addition, the mixture was allowed to warm to room temperature and stirred for 2.5 h. After completion of hydroboration 0.5 mL of cold water was added and the mixture was stirred for 10 min. This was followed by adding 0.3 mL of 3 N aqueous solution of NaOH and then 0.3 mL of 35% H_2_O_2_. The mixture was stirred for an additional 30 min, then quenched by the addition of 5 mL ice-cold water and extracted with EtOAc (3 × 10 mL). The organic phases were collected, washed with brine, dried over Na_2_SO_4_ and evaporated. The residue was subjected to chromatography on silica gel (R_f_ = 0.21; n-hexane/EtOAc = 3/2).

Method B: to a cooled (0 °C) solution of sabinol **1** (0.15 g, 1 mmoL) in 5 mL dry THF under argon atmosphere 0.21 mL of 95% solution of borane dimethyl sulfide complex (BMS) in DMS was injected, then the reaction mixture was allowed to warm up to room temperature and stirred for 18 h. The work up of the reaction mixture was the same as for Method A.

Compound **11**: *Method A:* 0.08 g, (47%); *Method B:* 0.12 g (70%); colorless crystals; mp: 66–68 °C; ${\left[\alpha \right]}_{D}^{20}$ = +35 (*c* = 0.250; MeOH). ^1^H NMR (500 MHz, CDCl_3_) δ (ppm): 0.31–0.35 (1H, m), 0.85–0.89 (4H, m), 0.93 (3H, d, *J* = 6.8 Hz), 0.95–0.99 (1H, m), 1.37 (1H, septet, *J* = 6.8 Hz), 1.75 (1H, d, *J* = 14.1 Hz), 2.08 (1H, ddd, *J* = 1.4, 6.8, 14.2 Hz), 2.10 (1H, s), 2.46–2.53 (1H, m), 3.83 (1H, dd, *J* = 9.0, 10.6 Hz), 3.90 (1H, dd, *J* = 5.5, 10.7 Hz), 4.41 (1H, t, *J* = 6.5 Hz). ^13^C NMR (125.8 MHz, CDCl_3_) δ (ppm): 13.9, 19.8, 19.9, 24.2, 32.7, 33.0, 38.5, 47.7, 62.5, 73.3. Anal. calcd for C_10_H_18_O_2_: C 70.55; H 10.66. Found: C 70.48; H 10.79.

*Synthesis of (1R,3S,5R)-3-benzyloxy-1-isopropyl-4-methylenebicyclo[3.1.0]hexane* (**12**). To a stirred suspension of NaH (6 mmoL) in dry, freshly distilled THF (10 mL), a THF solution (3 mL) of **1** (0.30 g, 2 mmoL) was added at room temperature under argon atmosphere. After 30 min stirring, a solution of benzyl bromide (0.34 g, 2 mmoL) in THF (3 mL) was added dropwise at room temperature, and the reaction mixture was kept at boiling temperature for 1 h. The reaction was quenched by the addition of H_2_O (2 mL), and then THF was removed under reduced pressure to about 10% of the initial volume. After adding ice-cold water (25 mL) to the obtained residue, the aqueous phase was extracted with CH_2_Cl_2_ (4 × 20 mL), then the organic phase was dried (Na_2_SO_4_), filtered and evaporated to dryness. The crude residue was purified by flash column chromatography on silica gel (R_f_ = 0.80; n-hexane/EtOAc = 19:1).

Compound **12**: 0.45 g (92%); pale-yellow oil; ${\left[\alpha \right]}_{D}^{20}$ = +7 (*c* = 0.250; MeOH). ^1^H NMR (500 MHz, CDCl_3_) δ (ppm): 0.69–0.73 (1H, m), 0.87 (3H, d, *J* = 6.9 Hz), 0.93 (3H, d, *J* = 6.8 Hz), 1.18–1.21 (1H, m), 1.46 (1H, septet, *J* = 6.8 Hz), 1.64 (1H, dd, *J* = 2.4, 8.5 Hz), 1.89 (1H, d, *J* = 13.9 Hz), 2.05 (1H, ddd, *J* = 1.4, 7.3, 13.8 Hz), 4.08 (1H, d, *J* = 7.2 Hz), 4.32 (1H, d, *J* = 11.8 Hz), 4.57 (1H, d, *J* = 11.7 Hz), 4.88 (1H, s), 5.11 (1H, s), 7.27–7.39 (5H, m). ^13^C NMR (125.8 MHz, CDCl_3_) δ (ppm): 18.6, 19.7, 19.8, 29.1, 32.6, 35.7, 36.7, 69.8, 81.3, 108.4, 127.3, 127.7, 128.3, 138.7, 152.3. Anal. calcd for C_17_H_22_O: C 84.25; H 9.15. Found: C 84.43; H 9.33.

### 3.2. General Procedure for the Preparation of ***13*** and ***14***

To a solution of **1** or **12** (0.66 mmoL) in acetone (5 mL) 4-methylmorpholine *N*-oxide (0.33 mL, 50% aqueous solution) and OsO_4_ (0.25 mL, 2% *tert*-BuOH solution) were added, and the reaction mixture was stirred for 24 h at room temperature. Then, the reaction was quenched by the addition of saturated aqueous Na_2_SO_3_ (5 mL) and extracted with EtOAc (3 × 10 mL). The organic layer was dried (Na_2_SO_4_) and evaporated. The purification of the crude product was accomplished by column chromatography on silica gel (**13**: R_f_ = 0.15, **14**: R_f_ = 0.40; *n*-hexane/EtOAc = 3/2).

(*1R,2R,3S,5R)-2-Hydroxymethyl-5-isopropylbicyclo[3.1.0]hexane-2,3-diol* (**13**): 0.10 g (81%); white crystalline powder; mp: 148–150 °C; ${\left[\alpha \right]}_{D}^{20}$ = +4 (*c* = 0.250; MeOH). ^1^H NMR (500 MHz, (CD_3_)_2_SO) δ (ppm): 0.18–0.24 (1H, m), 0.82 (3H, d, *J* = 6.8 Hz), 0.84 (1H, t, *J* = 4.1 Hz), 0.90 (3H, d, *J* = 6.8 Hz), 0.96–1.01 (1H, m), 1.34 (1H, septet, *J* = 6.8 Hz), 1.46 (1H, d, *J* = 13.2 Hz), 2.09 (1H, ddd, *J* = 1.0, 6.6, 13.0 Hz), 3.42 (1H, dd, *J* = 6.3, 11.0 Hz), 3.55 (1H, dd, *J* = 5.4, 11.0 Hz), 3.69–3.73 (1H, m), 4.08 (1H, t, *J* = 5.8 Hz), 4.12 (1H, s), 4.42 (1H, d, *J* = 4.1 Hz). ^13^C NMR (125.8 MHz, (CD_3_)_2_SO) δ (ppm): 12.5, 19.8, 20.0, 30.0, 31.8, 32.3, 36.1, 63.8, 76.9, 83.4. Anal. calcd for C_10_H_18_O_3_: C 64.49; H 9.74. Found: C 64.60; H 9.88.

(*1R,2R,3S,5R)-3-Benzyloxy-2-hydroxymethyl-5-isopropylbicyclo[3.1.0]hexan-2-ol* (**14**): 0.08 g (44%); colorless semi-solid; ${\left[\alpha \right]}_{D}^{20}$ = +4 (*c* = 0.250; MeOH). ^1^H NMR (500 MHz, CDCl_3_) δ (ppm): 0.41–0.46 (1H, m), 0.90 (3H, d, *J* = 6.9 Hz), 0.94–0.97 (1H, m), 0.98 (3H, d, *J* = 6.8 Hz), 1.04–1.08 (1H, m), 1.44 (1H, septet, *J* = 6.8 Hz), 1.88 (1H, d, *J* = 13.9 Hz), 2.11 (1H, ddd, *J* = 1.6, 6.6, 13.9 Hz), 3.66 (1H, d, *J* = 11.4 Hz), 3.73 (1H, d, *J* = 6.5 Hz), 3.94 (1H, d, *J* = 11.4 Hz), 4.27 (1H, d, *J* = 11.8 Hz), 4.57 (1H, d, *J* = 11.8 Hz), 7.26–7.37 (5H, m). ^13^C NMR (125.8 MHz, CDCl_3_) δ (ppm): 13.2, 19.9, 20.1, 29.3, 31.7, 32.2, 33.3, 65.2, 71.4, 85.1, 85.8, 127.5, 127.7, 128.5, 138.2. Anal. calcd for C_17_H_24_O_3_: C 73.88; H 8.75. Found: C 73.99; H 8.84.

### 3.3. General Procedure for the Preparation of ***17*** and ***18***

*Method A:* to a mixture of **1** (1.00 g, 6.57 mmoL) dissolved in 50 mL CH_2_Cl_2_ and Na_2_HPO_4_·12H_2_O (3.40 g, 9.49 mmoL) dissolved in 100 mL water (pH = 9.2), *m*-chloroperbenzoic acid (75% purity, 7.47 mmoL) was added in one portion at 0 °C, and the mixture was stirred at room temperature. When the reaction was complete, as indicated by TLC (1 h), the mixture was separated, and the aqueous phase was extracted with CH_2_Cl_2_ (100 mL). The organic layer was washed with 10% NaOH solution (3 × 50 mL), dried (Na_2_SO_4_), and evaporated. The residue was purified by column chromatography on silica gel to afford a mixture of **17** and **18** (0.78 g, 37%, **17**:**18** = 2:1). Product **17** (0.30 g) was isolated in pure form by recrystallization from CH_2_Cl_2_/*n*-hexane.

*Method B:* to a solution of **1** (0.50 g, 3.28 mmoL) in 25 mL CH_2_Cl_2_ and Na_2_HPO_4_·12H_2_O (1.70 g, 4.74 mmoL) in 50 mL water (pH = 9.2), a solution of *m*-chloroperbenzoic acid (75% purity, 3.74 mmoL) in CH_2_Cl_2_ (25 mL) was added dropwise over 10 min at 0 °C, and the mixture was stirred at room temperature. When the reaction was complete, as indicated by TLC (1 h), the mixture was separated, and the aqueous phase was extracted with CH_2_Cl_2_ (50 mL). The organic layer was washed with saturated aqueous solution of NaHCO_3_ (3 × 50 mL), dried (Na_2_SO_4_), and evaporated to afford **18** as the single product.

*((1R,2S,3S,5R)-2,3-dihydroxy-5-isopropylbicyclo[3.1.0]hexan-2-yl)methyl 3-chlorobenzoate* (**17**). Method A, 0.30 g (14%, in isolated pure form); white crystals; mp: 93–96 °C; ${\left[\alpha \right]}_{D}^{20}$ = +22 (*c* = 0.250; MeOH). ^1^H NMR (500 MHz, CDCl_3_) δ (ppm): 0.48–0.52 (1H, m), 0.87 (3H, d, *J* = 6.9 Hz), 0.92 (3H, d, *J* = 6.8 Hz), 1.16 (1H, t, *J* = 4.2 Hz), 1.30–1.37 (2H, m), 1.89 (1H, d, *J* = 14.3 Hz), 2.10 (1H, ddd, *J* = 1.1, 7.0, 14.2 Hz), 2.54 (1H, br s), 2.81 (1H, br s), 3.95 (1H, d, *J* = 7.0 Hz), 4.30 (1H, d, *J* = 11.6 Hz), 4.39 (1H, d, *J* = 11.6 Hz), 7.40 (1H, t, *J* = 7.9 Hz), 7.55 (1H, d, *J* = 8.0 Hz), 7.96 (1H, d, *J* = 7.9 Hz), 8.04 (1H, s). ^13^C NMR (125.8 MHz, CDCl_3_) δ (ppm): 13.7, 19.5, 19.7, 29.5, 32.4, 32.8, 35.2, 69.4, 72.9, 80.7, 127.9, 129.8, 129.8, 131.5, 133.3, 134.7, 165.6. Anal. calcd for C_17_H_21_ClO_4_: C 62.86; H 6.52. Found: C 63.01; H 6.60.

*(1R,3S,4S,5R)-4-hydroxy-4-hydroxymethyl-1-isopropylbicyclo[3.1.0]hexan-3-yl 3-chlorobenzoate* (**18**)*.* Method B, 0.40 g (38%); white crystalline powder; mp: 110–112 °C; ${\left[\alpha \right]}_{D}^{20}$ = +12 (*c* = 0.250; MeOH). ^1^H NMR (400 MHz, CDCl_3_) δ (ppm): 0.55–0.61 (1H, m), 0.91 (3H, d, *J* = 6.9 Hz), 0.95 (3H, d, *J* = 6.8 Hz), 1.23 (1H, t, *J* = 4.4 Hz), 1.38 (1H, septet, *J* = 6.8 Hz), 1.75 (1H, dd, *J* = 3.7, 8.6 Hz), 1.87 (1H, d, *J* = 14.1 Hz), 2.16 (1H, ddd, *J* = 1.5, 7.2, 14.2 Hz), 2.19 (1H, br s), 3.03 (1H, br s), 3.83 (1H, d, *J* = 12.6 Hz), 3.96 (1H, d, *J* = 12.6 Hz), 4.37 (1H, d, *J* = 7.0 Hz), 7.40 (1H, t, *J* = 7.9 Hz), 7.55 (1H, d, *J* = 8.0 Hz), 7.91 (1H, d, *J* = 7.8 Hz), 8.00 (1H, s). ^13^C NMR (125.8 MHz, CDCl_3_) δ (ppm): 15.1, 19.9, 20.0, 27.7, 32.9, 33.2, 35.2, 66.9, 73.7, 94.0, 128.3, 130.2, 132.6, 133.8, 134.3, 135.1, 166.8. Anal. calcd for C_17_H_21_ClO_4_: C 62.86; H 6.52. Found: C 62.97; H 6.75.

*Synthesis of (1R,2S,3S,5R)-2-hydroxymethyl-5-isopropylbicyclo[3.1.0]hexane-2,3-diol* (**19**). To a solution of **17** or **18** (33 mg, 0.10 mmoL) in EtOH (2 mL), a 10% cold aqueous solution of NaOH (1 mL) was added at room temperature. The reaction mixture was stirred for 30 min, then concentrated under reduced pressure to evaporate ethanol, and the aqueous residue was extracted with CH_2_Cl_2_ (3 × 3 mL). The combined organic extract was washed with brine, dried (Na_2_SO_4_) and evaporated. 

Compound **19**: 17 mg (91%); white crystalline powder; mp: 90–92 °C; ${\left[\alpha \right]}_{D}^{20}$ = +45 (*c* = 0.250; MeOH). ^1^H NMR (500 MHz, (CD_3_)_2_SO) δ (ppm): 0.22–0.26 (1H, m), 0.80 (3H, d, *J* = 6.8 Hz), 0.85 (3H, d, *J* = 6.8 Hz), 0.94 (1H, t, *J* = 3.9 Hz), 1.03 (1H, dd, *J* = 3.5, 8.5 Hz), 1.23 (1H, septet, *J* = 6.8 Hz), 1.55 (1H, d, *J* = 13.6 Hz), 1.89 (1H, ddd, *J* = 1.0, 7.1, 13.6 Hz), 3.21 (1H, dd, *J* = 6.4, 11.1 Hz), 3.33 (1H, dd, *J* = 5.6, 11.1 Hz), 3.64–3.68 (1H, m), 3.95 (1H, s), 4.44 (1H, d, *J* = 3.8), 4.55 (1H, t, *J* = 5.9 Hz). ^13^C NMR (125.8 MHz, (CD_3_)_2_SO) δ (ppm): 13.2, 19.4, 19.5, 29.2, 31.2, 32.0, 35.0, 66.8, 71.1, 80.8. Anal. calcd for C_10_H_18_O_3_: C 64.49; H 9.74. Found: C 64.69; H 9.83.

### 3.4. General Procedure for the Reaction of Aldehydes with Diethylzinc in the Presence of Chiral Catalyst

To the respective catalyst (0.1 mmoL), 1 M Et_2_Zn in *n*-hexane solution (3 mL, 3 mmoL) was added under an Ar atmosphere at room temperature. The reaction was stirred for 25 min at room temperature (see Table 1), and benzaldehyde (1 mmoL) was then added to the solution with subsequent stirring at room temperature (see Table 1) for a further 20 h. The reaction was quenched with a saturated NH_4_Cl solution (15 mL), and the mixture was extracted with EtOAc (2 × 20 mL). The combined organic phase was washed with H_2_O (10 mL), dried (Na_2_SO_4_), and evaporated under vacuum. The crude secondary alcohols obtained were purified by flash column chromatography (R_f_ = 0.54; *n*-hexane/EtOAc = 4/1). The *ee* and absolute configuration of the resulting material were determined by chiral HPLC analysis on a Chiralcel OD-H column and the data are as follows: 1-phenyl-1-propanol; *V*(*n*-hexane)/*V*(2-propanol) = 95: 5, 0.7 mL/min, 215 nm, *t*_R1_ = 6.29 min for *R*-isomer, *t*_R2_ = 6.73 min for *S*-isomer [7].

### 3.5. X-ray Structure Determination of ***11***

Single crystals of 11 were grown by warming up 11 in heptane until almost full dissolution, and then the solution was abruptly cooled in an ice bath. X-ray intensity data were collected at –123 °C on a Rigaku UltraX 18S generator (Xenocs mirrors, Mo Kα radiation, λ = 0.71073 Å) using a MAR345 image plate. The images were interpreted and integrated with CrysAlisPRO [48], and the implemented absorption correction was applied. Using Olex2 [49], the structures were solved with the ShelXS [50] structure solution program by Direct Methods, and refined with the ShelXL [51] refinement package using full-matrix least-squares minimization on *F*^2^. Non-hydrogen atoms were refined anisotropically, and hydrogen atoms in the riding mode with isotropic temperature factors fixed at 1.2 times U_eq_ of the parent atoms (1.5 for the –CH_3_ and the –OH groups). Because compound 11 is composed of only light atoms (C, H, O), it was not possible to determine its absolute configuration. CCDC number 1814013 contains the supplementary crystallographic data.

Crystal Data for C_10_H_18_O_2_ (*M* = 170.24 g/moL): orthorhombic, space group *P*2_1_2_1_2_1_ (no. 19), *a* = 6.1474(2) Å, *b* = 8.1525(3) Å, *c* = 19.6783(6) Å, *V* = 986.21(6) Å^3^, *Z* = 4, *T* = 150.15 K, μ(MoKα) = 0.078 mm^−1^, *Dcalc* = 1.147 g/cm^3^, 8206 reflections measured (10.216° ≤ 2θ ≤ 52.006°), 1915 unique (*R*_int_ = 0.0310, R_sigma_ = 0.0189) which were used in all calculations. The final *R*_1_ was 0.0328 (I > 2σ(I)) and *wR*_2_ was 0.0848 (all data).

## 4. Conclusions

In conclusion, we have developed a library of new chiral sabinol-based aminodiol**,** diol, and triol derivatives (**6**–**19**). Our results revealed that functionalization of (+)-sabinol occurred with high stereoselectivity, affording only a single diastereomeric product in each case. Stereoselectivities, however, were found to be lower as compared with other monoterpene-based trifunctional catalysts. It is surmised that, because of the appreciable steric influence of the bicyclic ring system and the freely rotating hydroxymethyl or aminomethyl group, a stable transition state could not be formed in the reactions of the applied catalysts and Et_2_Zn. From this point of view, aminodiols have proven to be more efficient catalysts compared to diol or triol derivatives.

On the other hand, because of the observed different reactivity of the functional groups (caused by the steric hindrance of the bicyclic system of sabinane skeleton), the obtained aminodiols, diols, and triols may serve as useful building blocks for the synthesis of new heterocyclic ring systems and biologically active compounds.

## Figures and Tables

**Table 1:** Addition of diethylzinc to benzaldehyde, catalyzed by various types of diol, triols, and aminodiols at room temperature.

Entry	Catalyst (10 mol %)	Yield ^1^ (%)	*ee* ^2^ (%)	Config. of Major Product ^3^
1	**8**	88	44	*S*
2	**9**	98	66	*S*
3	**10**	98	58	*S*
4	**11**	73	6	*S*
5	**13**	88	12	*S*
6	**14**	87	17	*S*
7	**17**	73	9	*S*
8	**18**	95	4	*S*
9	**19**	81	8	*S*

^1^ Yields after silica column chromatography. ^2^ Determined on the crude product by HPLC (Chiracel OD–H). ^3^ Determined by comparing the *t*_R_ of the HPLC analysis and the optical rotation with the literature data [7].

## Supplementary Materials

The following are available online at http://www.mdpi.com/1420-3049/23/4/771/s1, ^1^H, ^13^C NMR, HSQC, HMBC, COSY, and NOESY spectra of new compounds (S3–S24) and crystal structure of diol **11** (S25).
