# Supplementary material for: Stereoselective Syntheses and Application of Chiral Bi- and Tridentate Ligands Derived from (+)-Sabinol
Source: Molecules. 2018 Mar 27;23(4):771. doi: 10.3390/molecules23040771 (PMC6017647; doi:10.3390/molecules23040771)

Supporting informations

for

Stereoselective syntheses and application of chiral bi- and tridentate ligands derived from (+)-sabinol

Yerbolat Tashenov[1], Mathias Daniels[2], Koen Robeyns[3], Luc Van Meervelt[2], Wim Dehaen[2], Yerlan M. Suleimen[1] Zsolt Szakonyi[4,5*]

[1] Institute of Applied Chemistry, Chemistry Department of L.N. Gumilyov Eurasian National University, Munaitpassov st., 5, 010008 Astana, the Republic of Kazakhstan

[2] KU Leuven, Department of Chemistry, Celestijnenlaan 200F, B-3001 Leuven, Belgium

[3] IMCN, Molecules Solids and Reactivity division (MOST), Université catholique de Louvain, Place Pasteur 1, B-1348 Louvain-la-Neuve, Belgium

[4] Institute of Pharmaceutical Chemistry, University of Szeged, H-6720 Szeged, Eötvös u. 6, Hungary

[5] Interdisciplinary Centre of Natural Products, University of Szeged, Szeged, Hungary

[*] Corresponding author. Tel.: +36-62-546809; Fax: +36-62-545705; szakonyi@pharm.u-szeged.hu

Contents

^{1}H, ^{13}C NMR, HSQC, HMBC, COSY and NOESY spectra of new compounds S3-S24

Crystal structure of diol **11** S25

2,2,2-Trichloro-*N*-(((1*R*,5*S*)-5-isopropylbicyclo[3.1.0]hex-2-en-2-yl)methyl)acetamide (**3**)

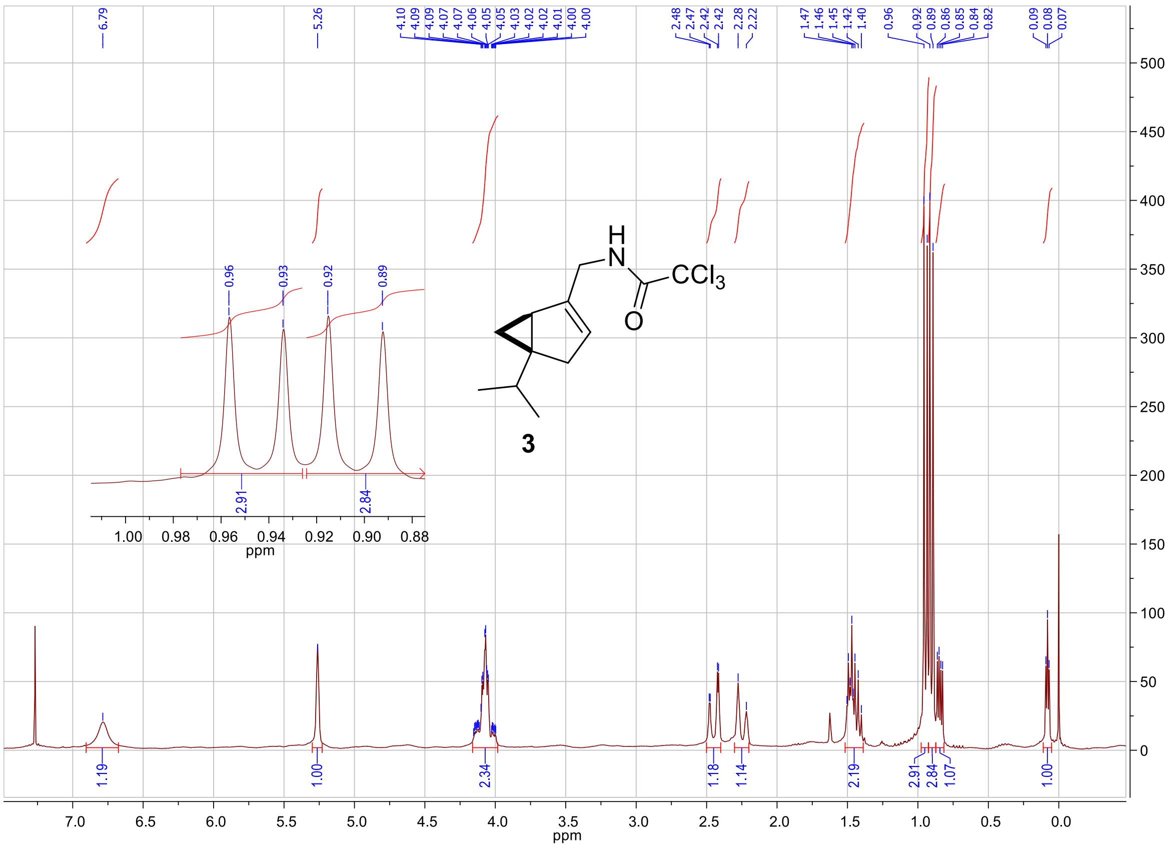

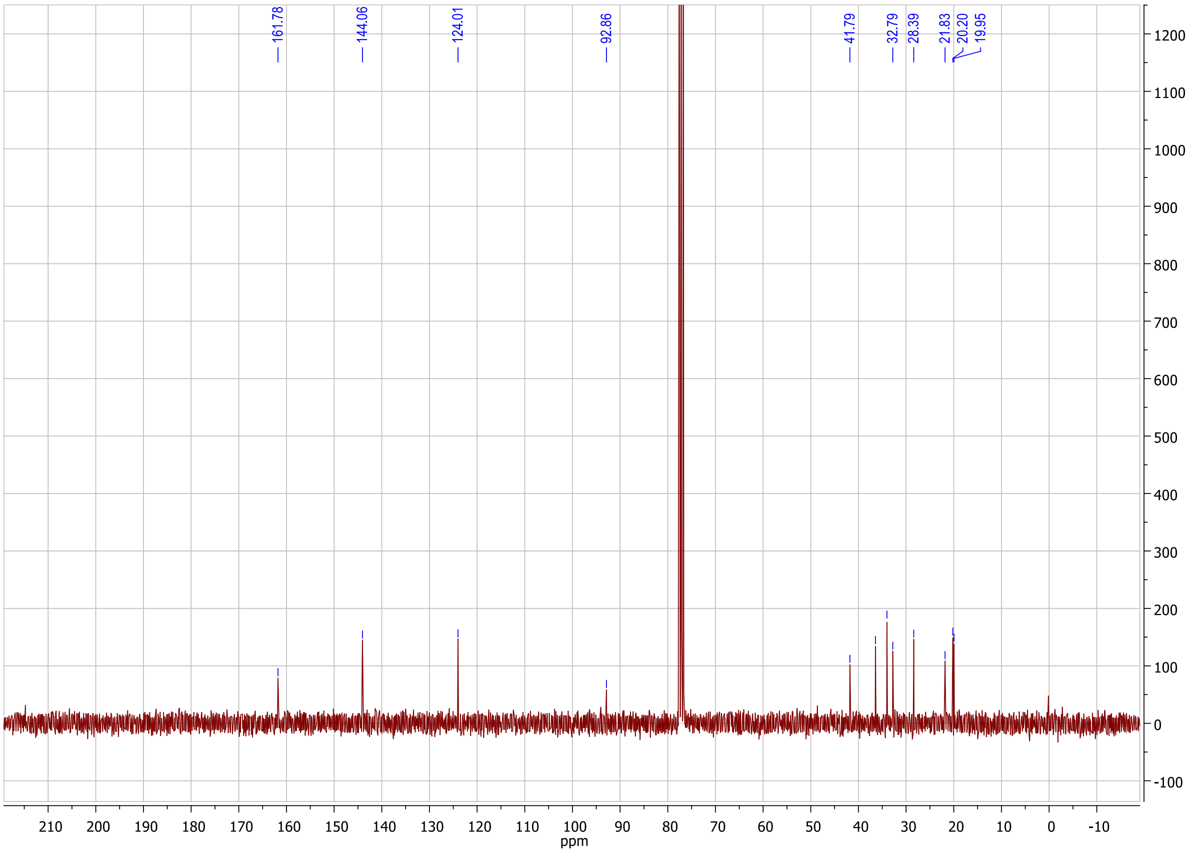

((1*R*,5*S*)-5-Isopropylbicyclo[3.1.0]hex-2-en-2-yl)methanamine (**4**)

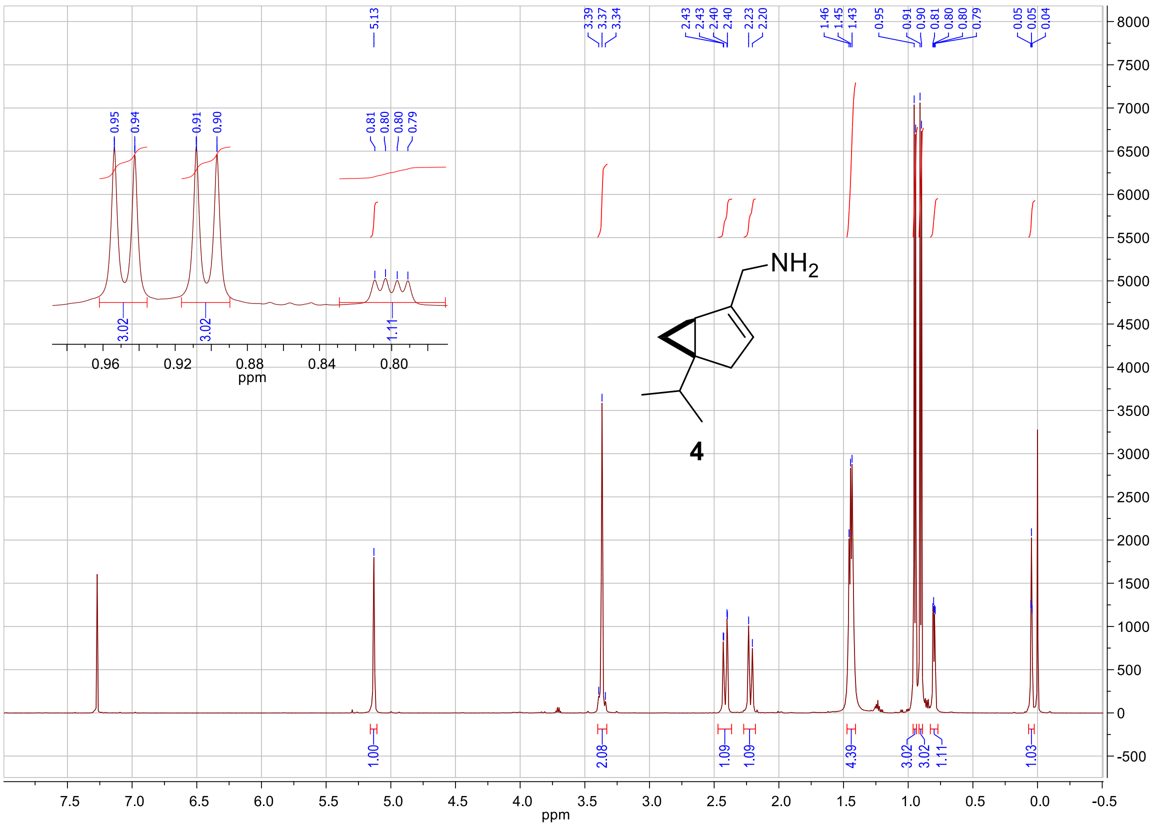

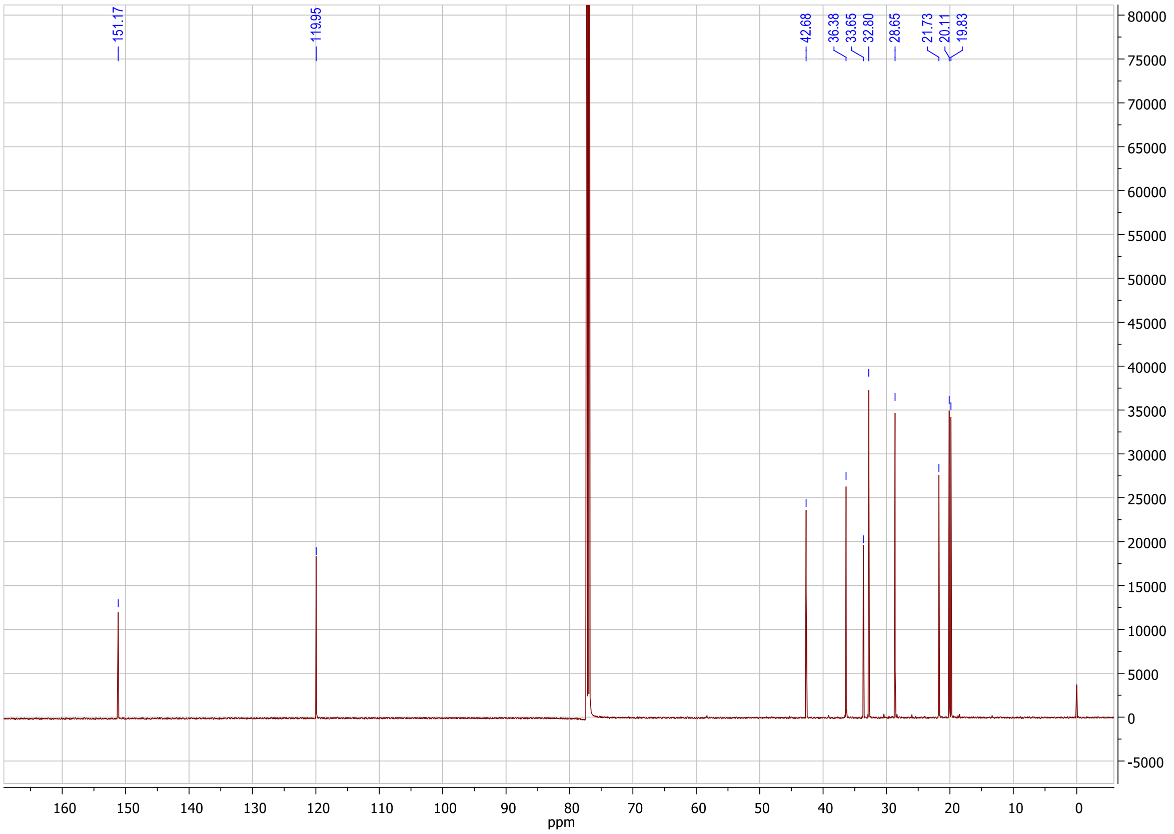

Tert-butyl (((1R,5S)-5-isopropylbicyclo[3.1.0]hex-2-en-2-yl)methyl)carbamate (**5**)

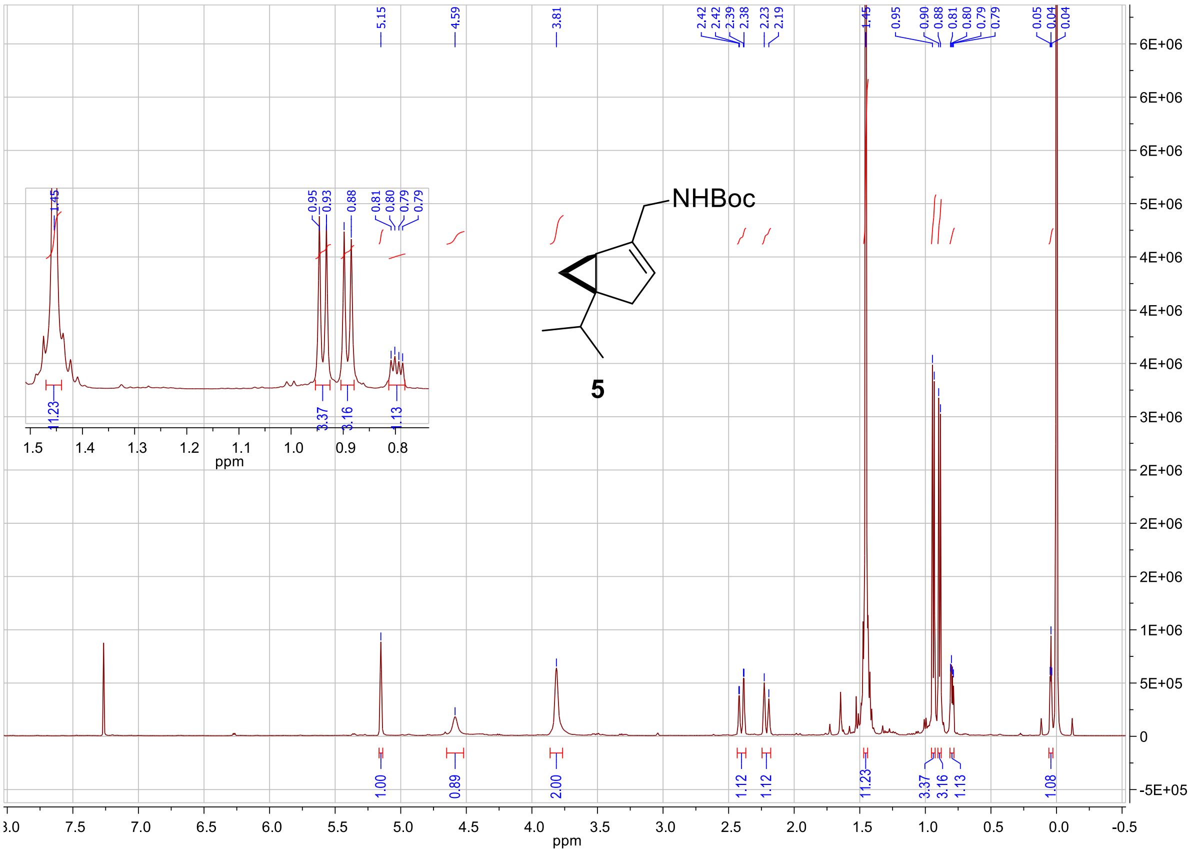

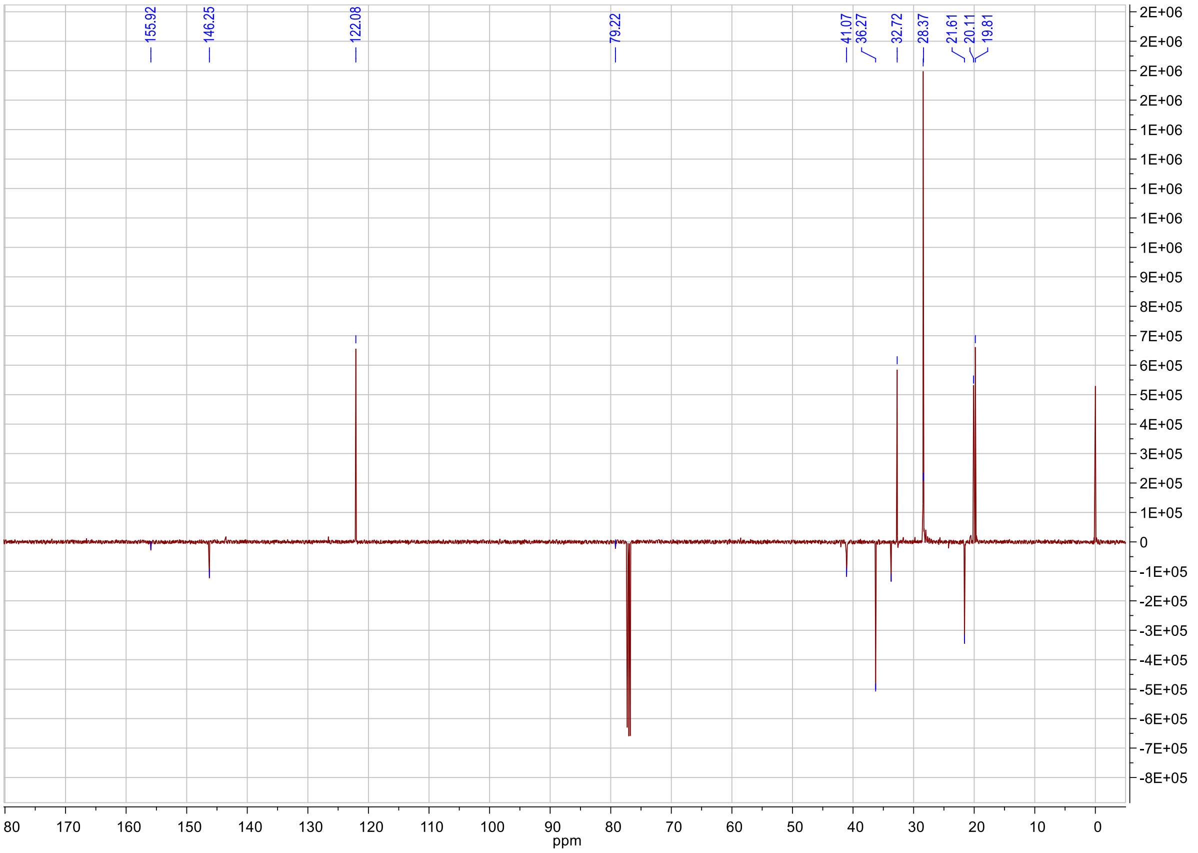

Tert-butyl (((1*R*,2*R*,3*R*,5*R*)-2,3-dihydroxy-5-isopropylbicyclo[3.1.0]hexan-2-yl)methyl)carbamate (**6**)

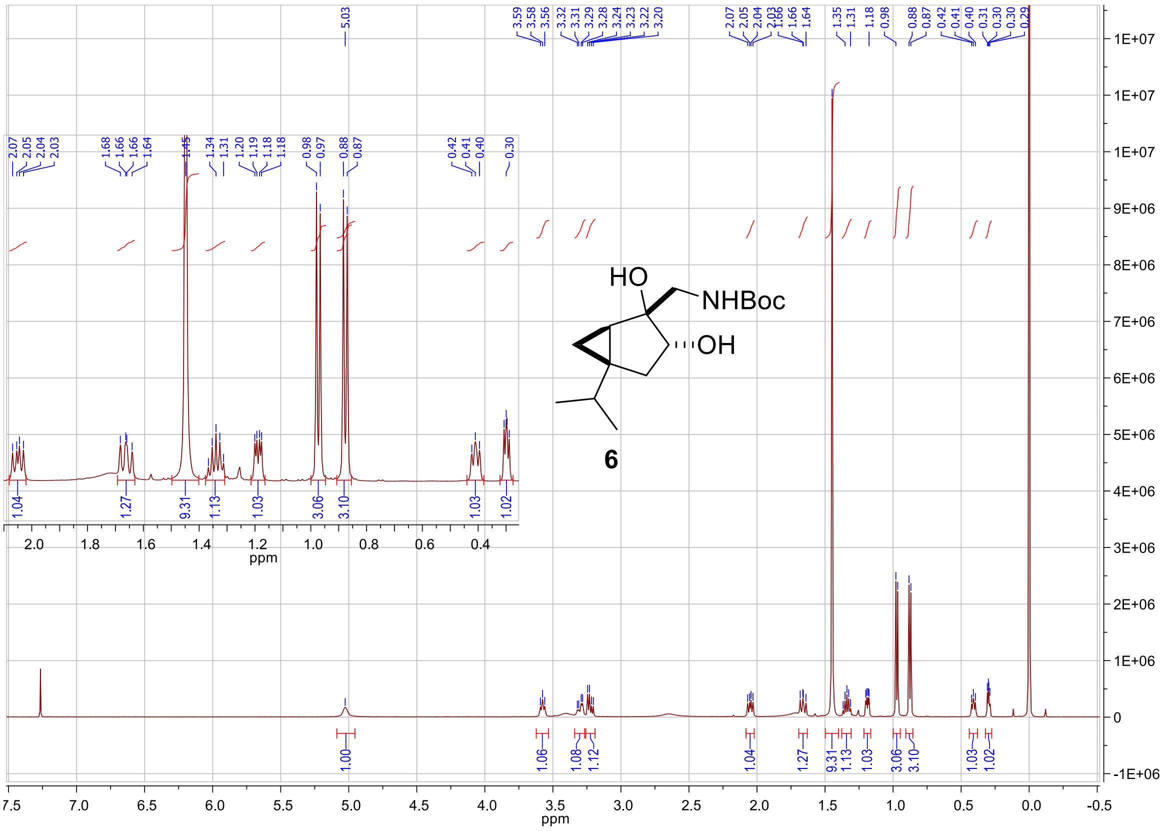

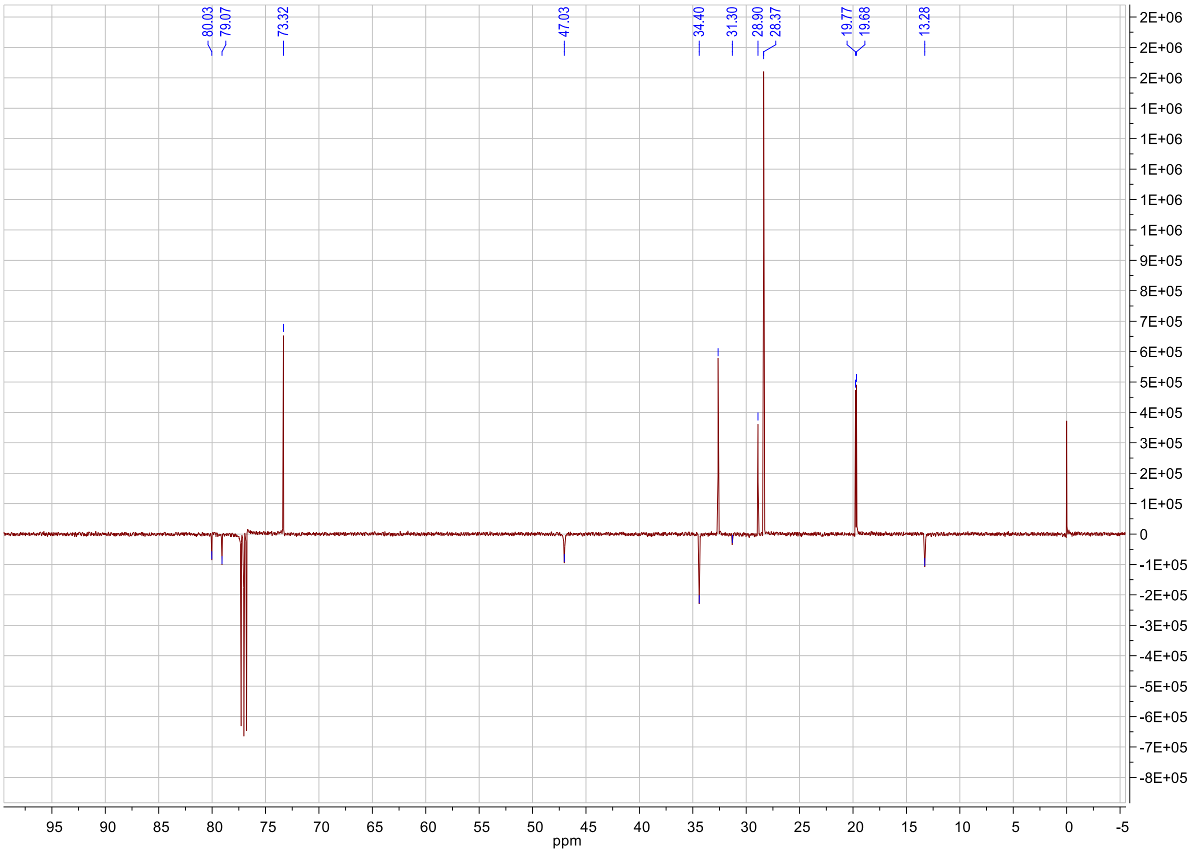

HMBC spectrum of **6**

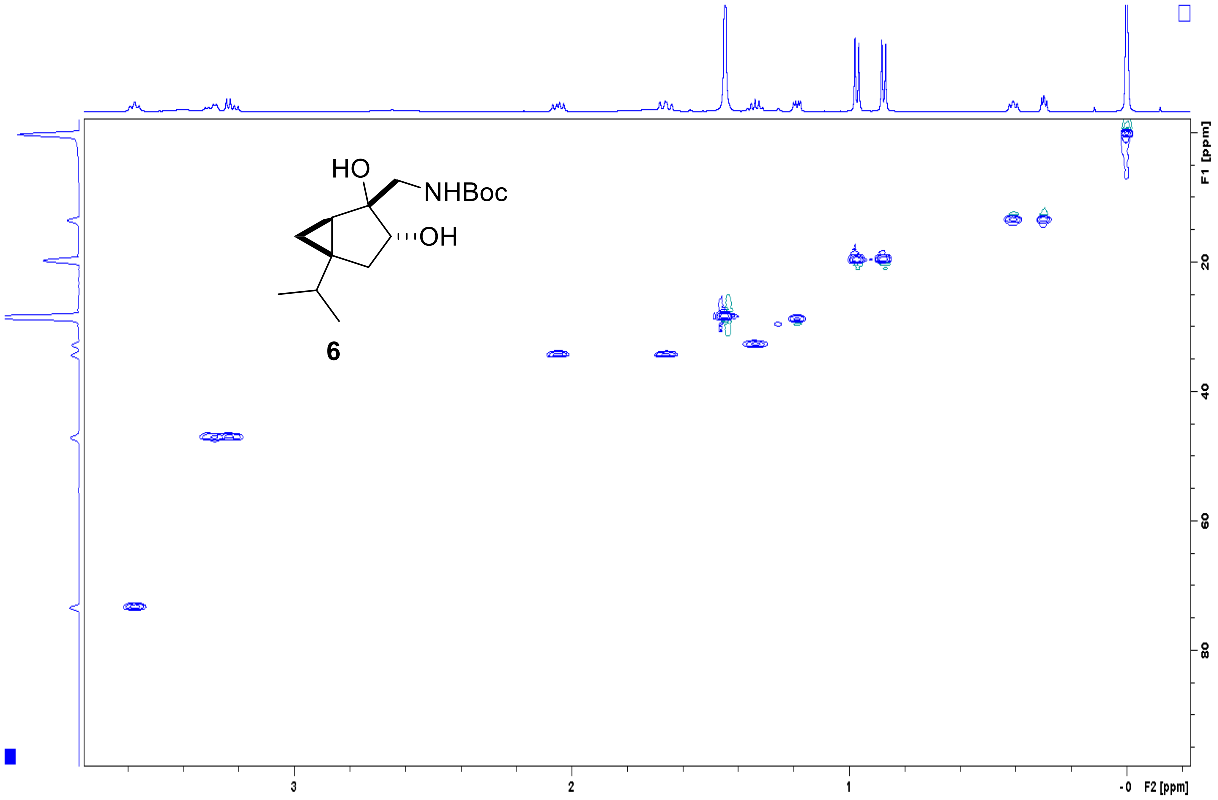

HSQC spectrum of **6**

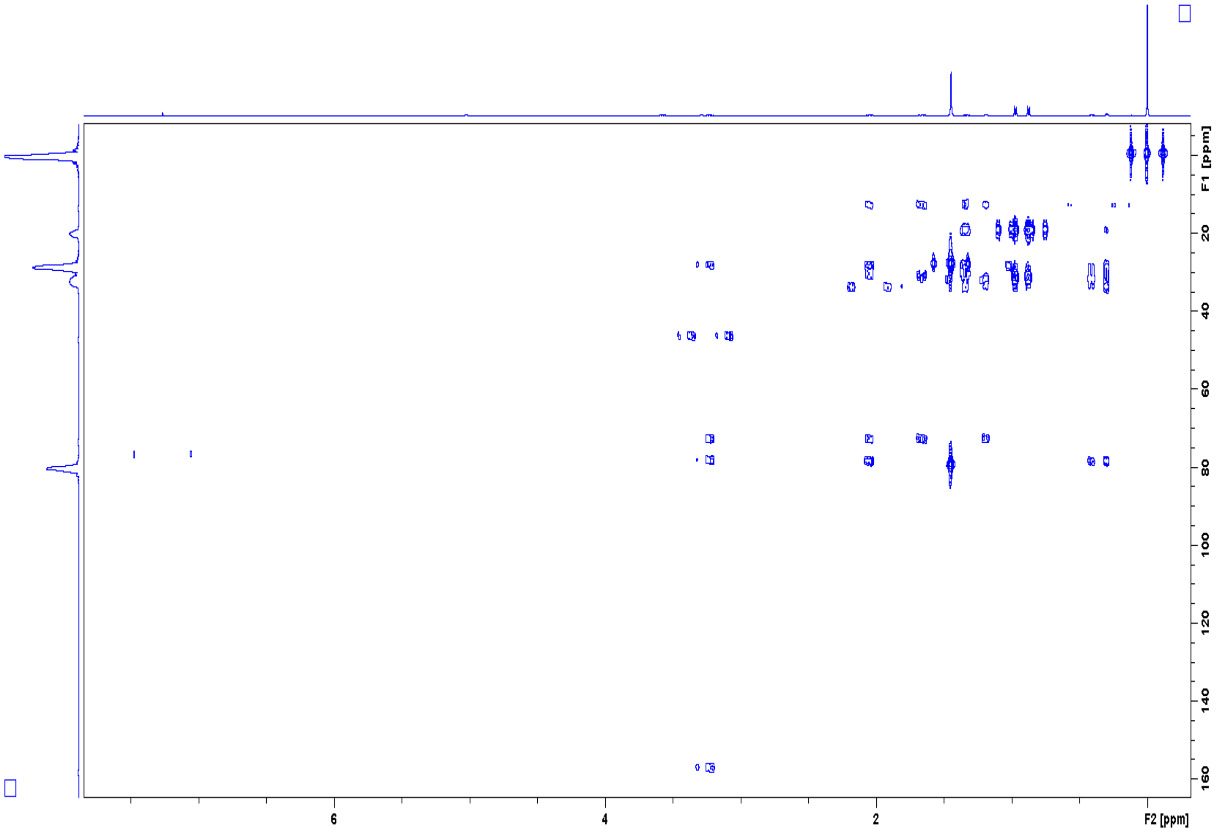

COSY spectrum of **6**

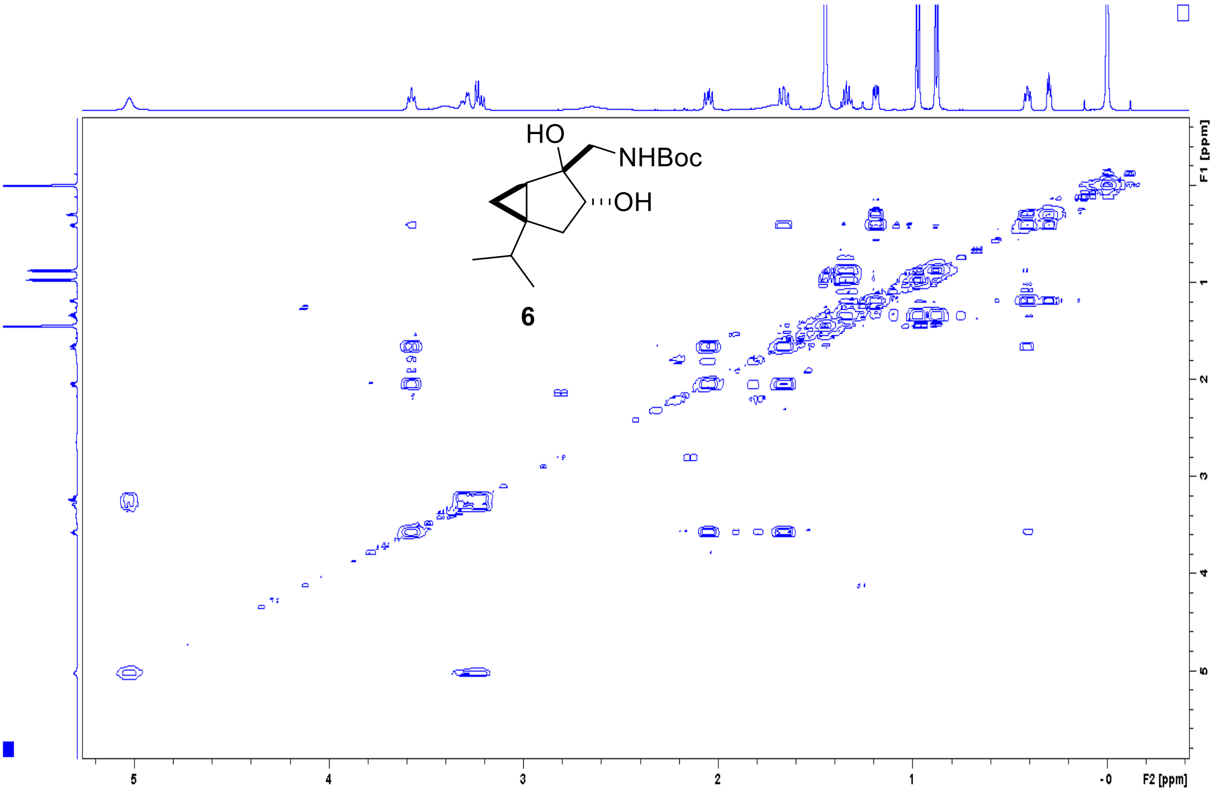

NOESY spectrum of **6**

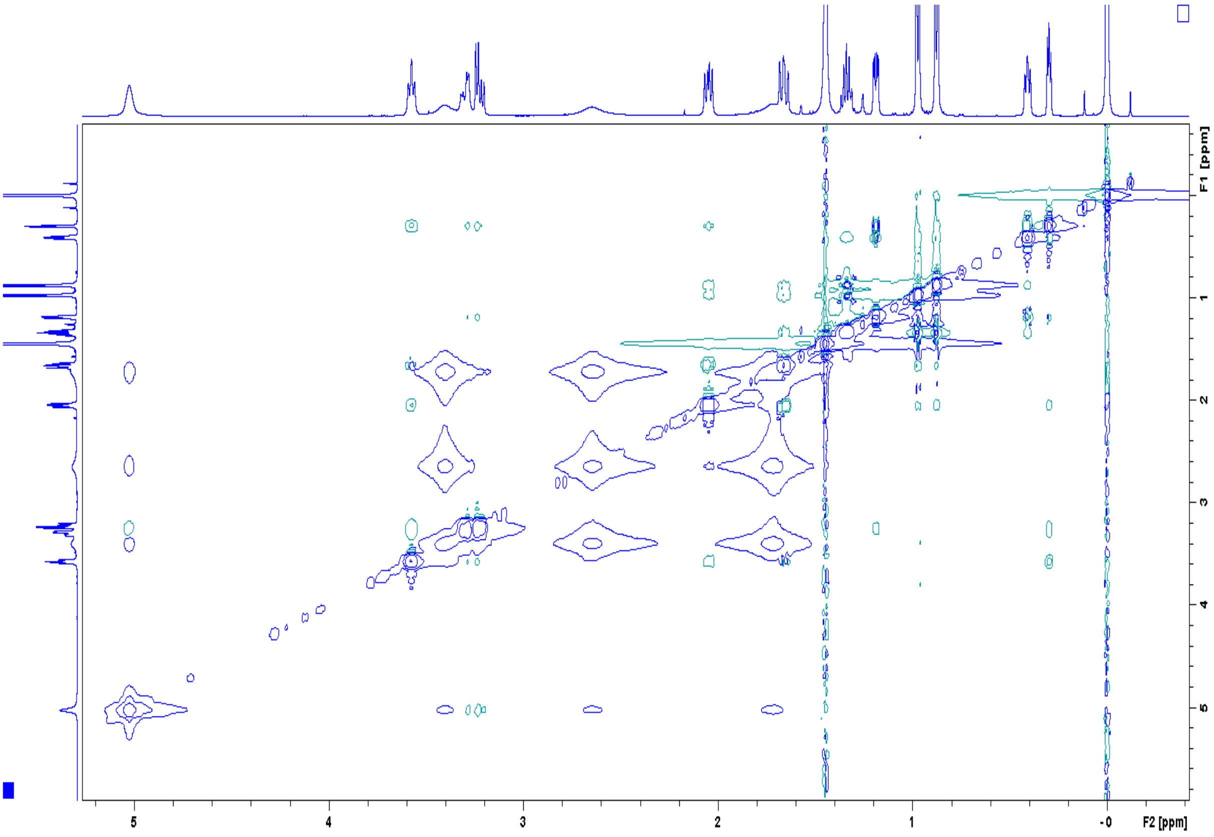

(1*R*,2*R*,3*R*,5*R*)-2-Aminomethyl-5-isopropylbicyclo[3.1.0]hexane-2,3-diol hydrochloride (**7**)

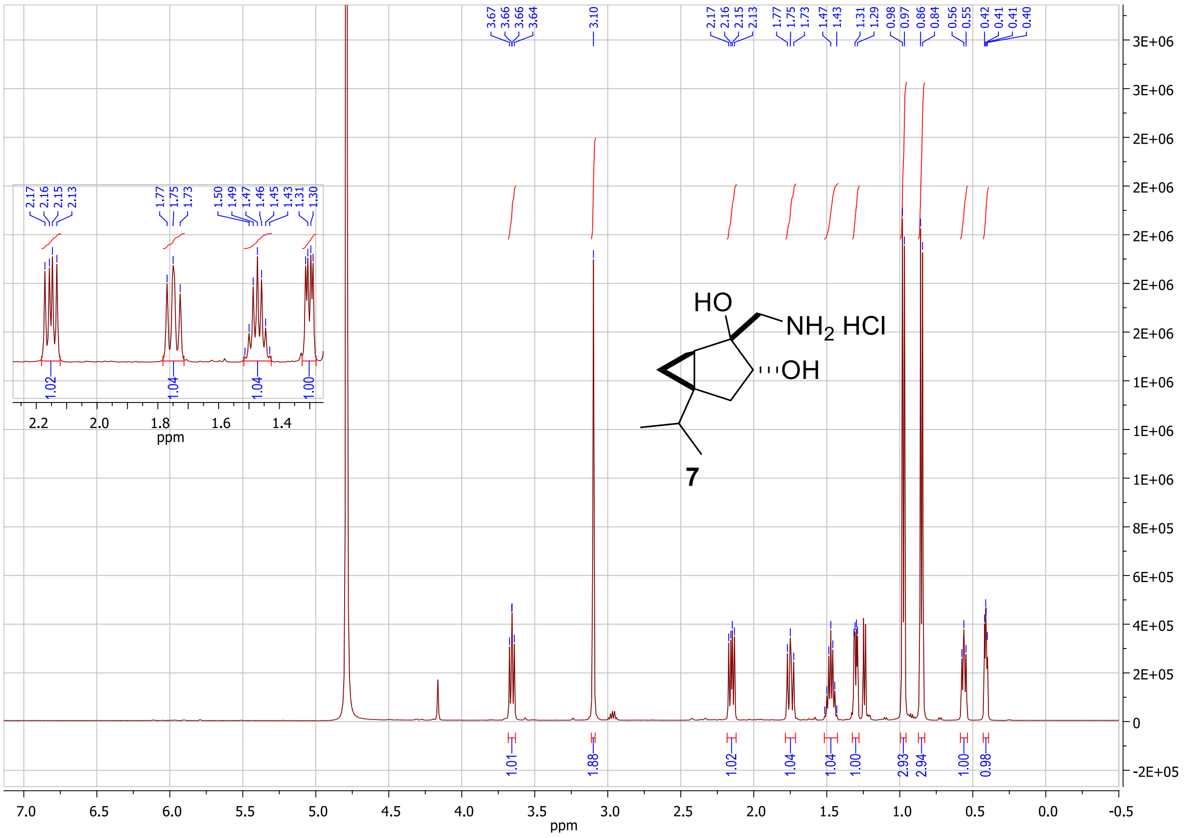

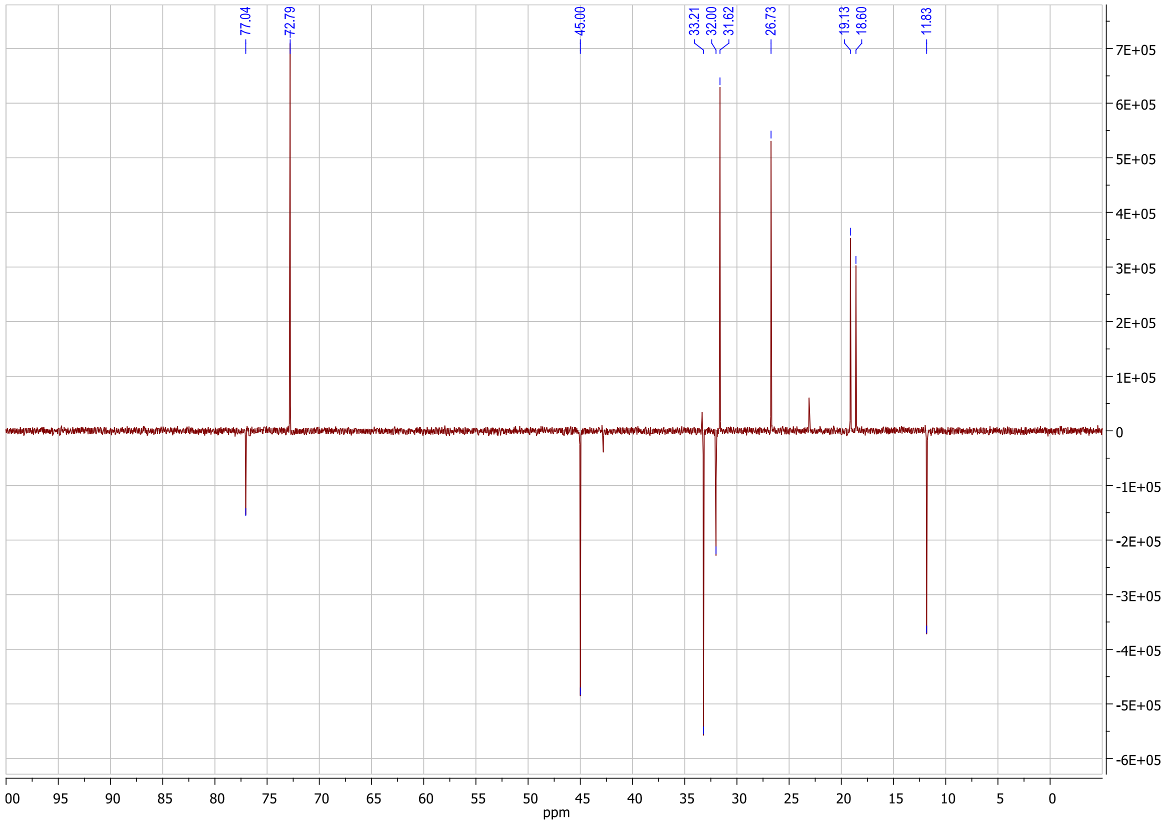

(1*R*,2*R*,3*R*,5*R*)-2-((Benzylamino)methyl)-5-isopropylbicyclo[3.1.0]hexane-2,3-diol (**9**)

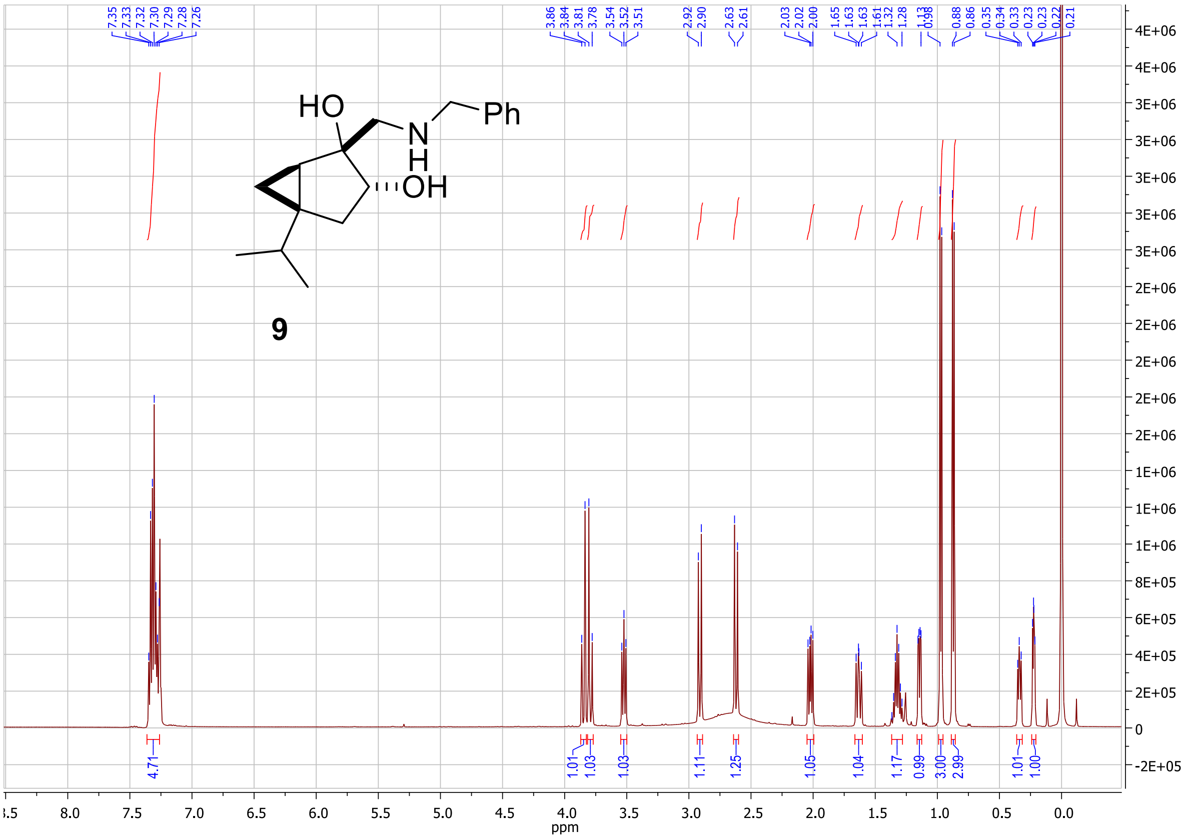

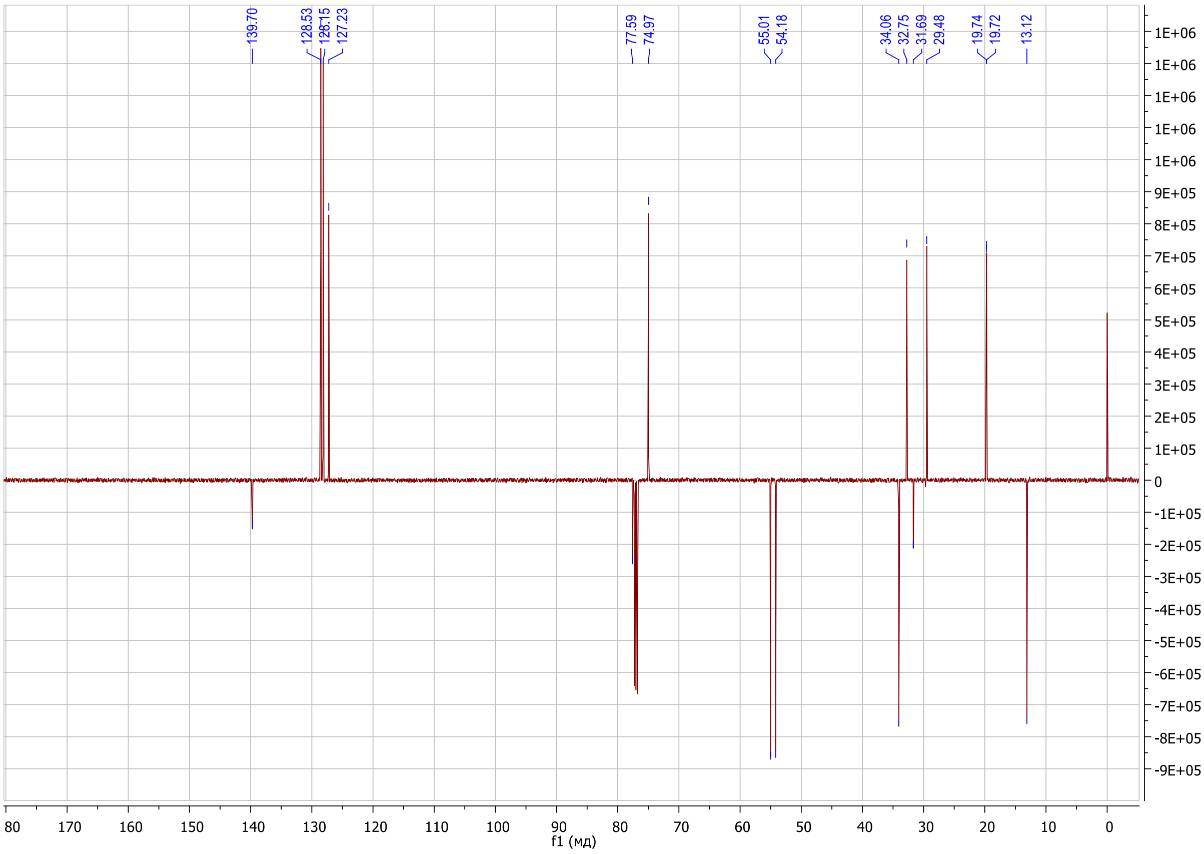

(1*R*,2*R*,3*R*,5*R*)-3'-Benzyl-5-isopropylspiro[bicyclo[3.1.0]hexane-2,5'-oxazolidin]-3-ol (**10**)

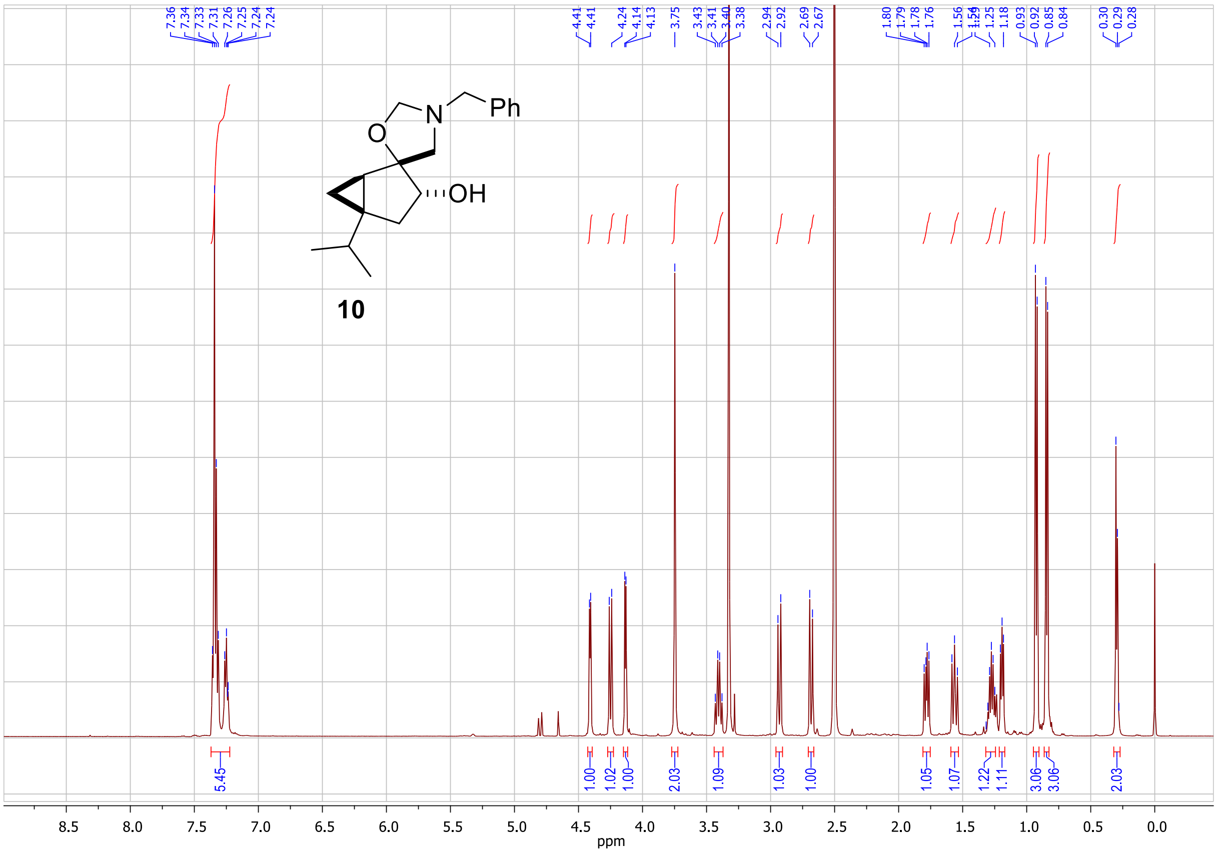

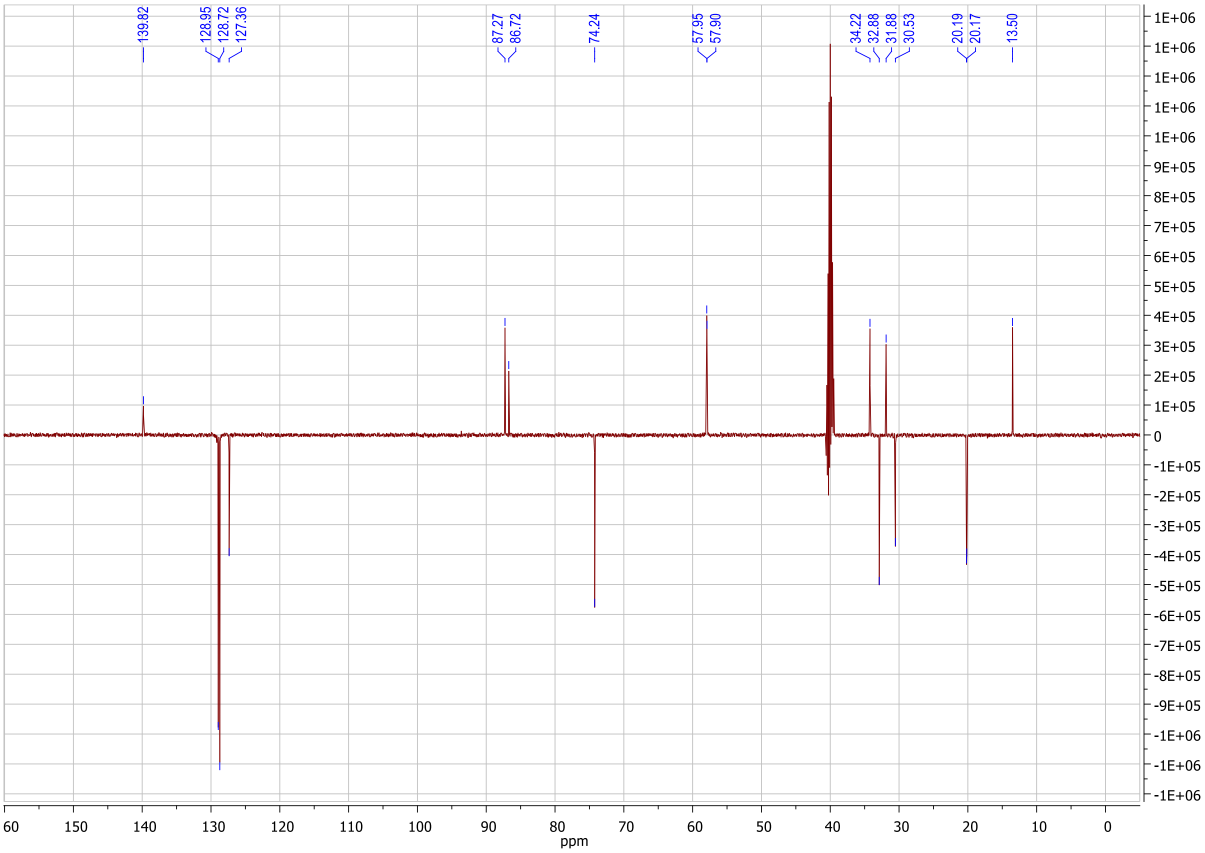

COSY spectrum of **10**

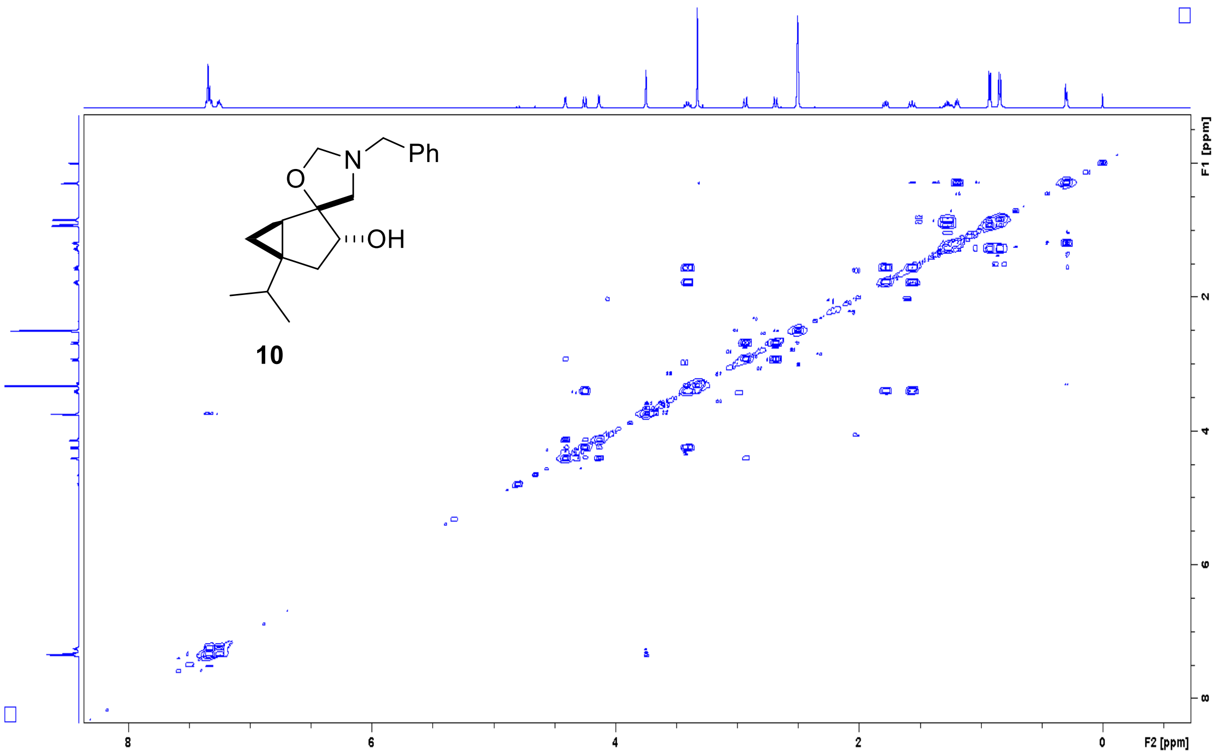

NOESY spectrum of **10**

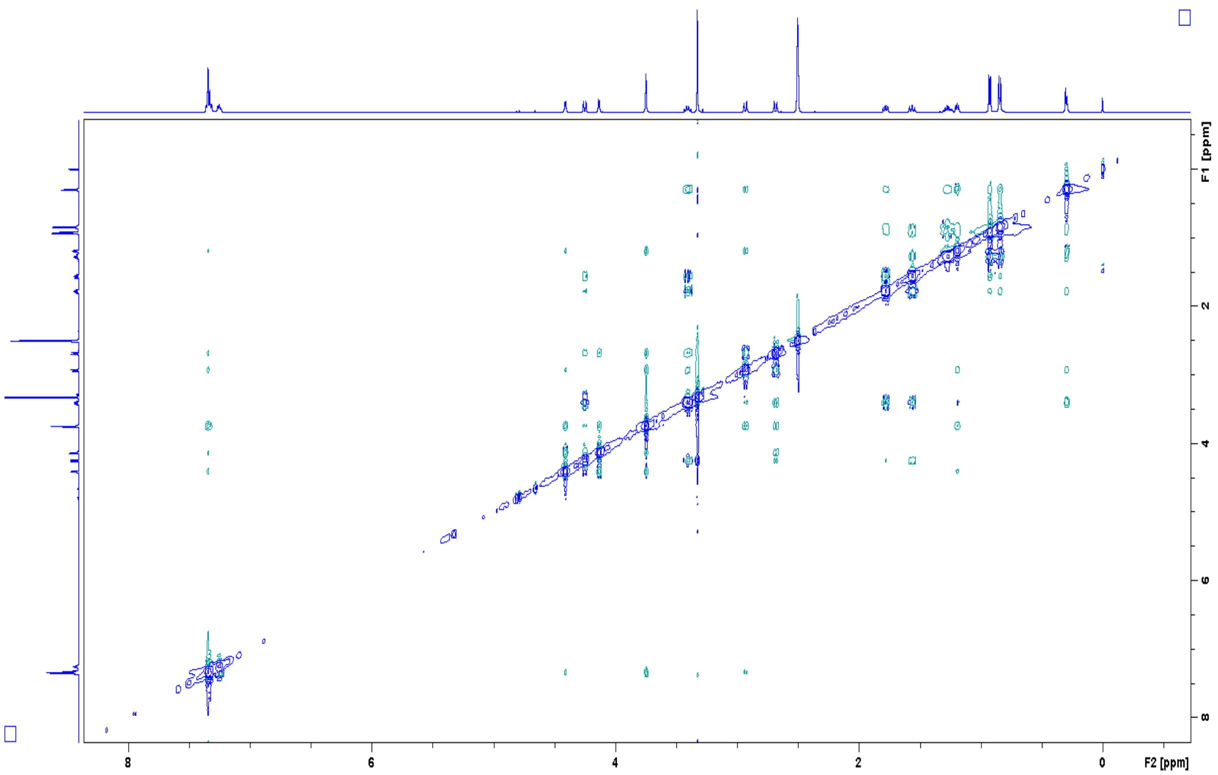

HSQC spectrum of **10**

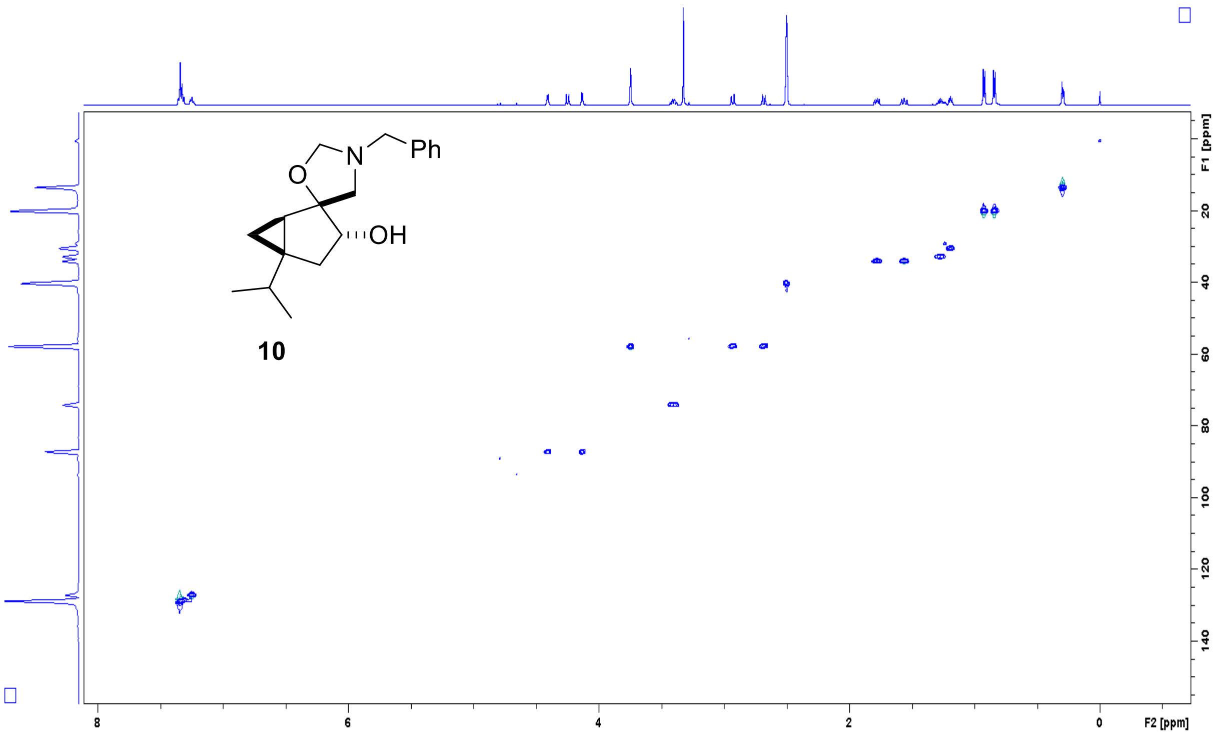

HMBC spectrum of **10**

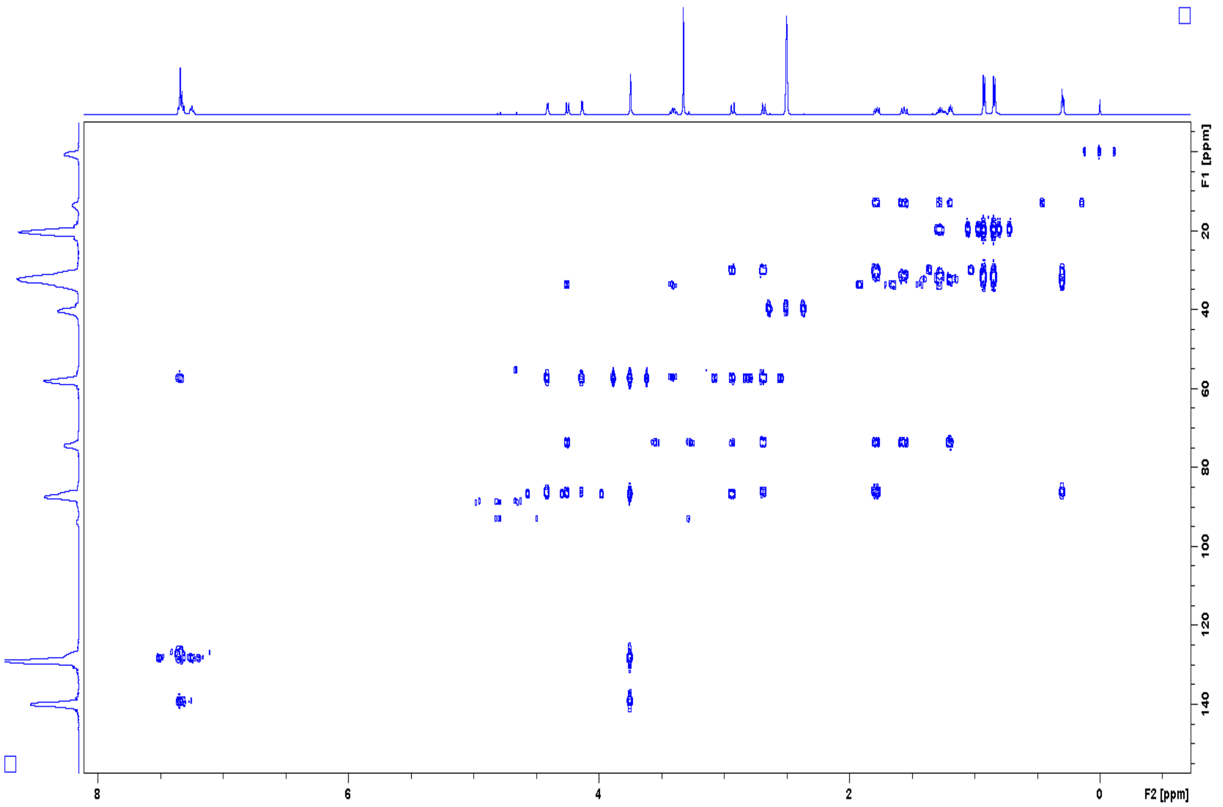

(1*R*,3*S*,4*S*,5*S*)-4-Hydroxymethyl-1-isopropylbicyclo[3.1.0]hexan-3-ol (**11**)

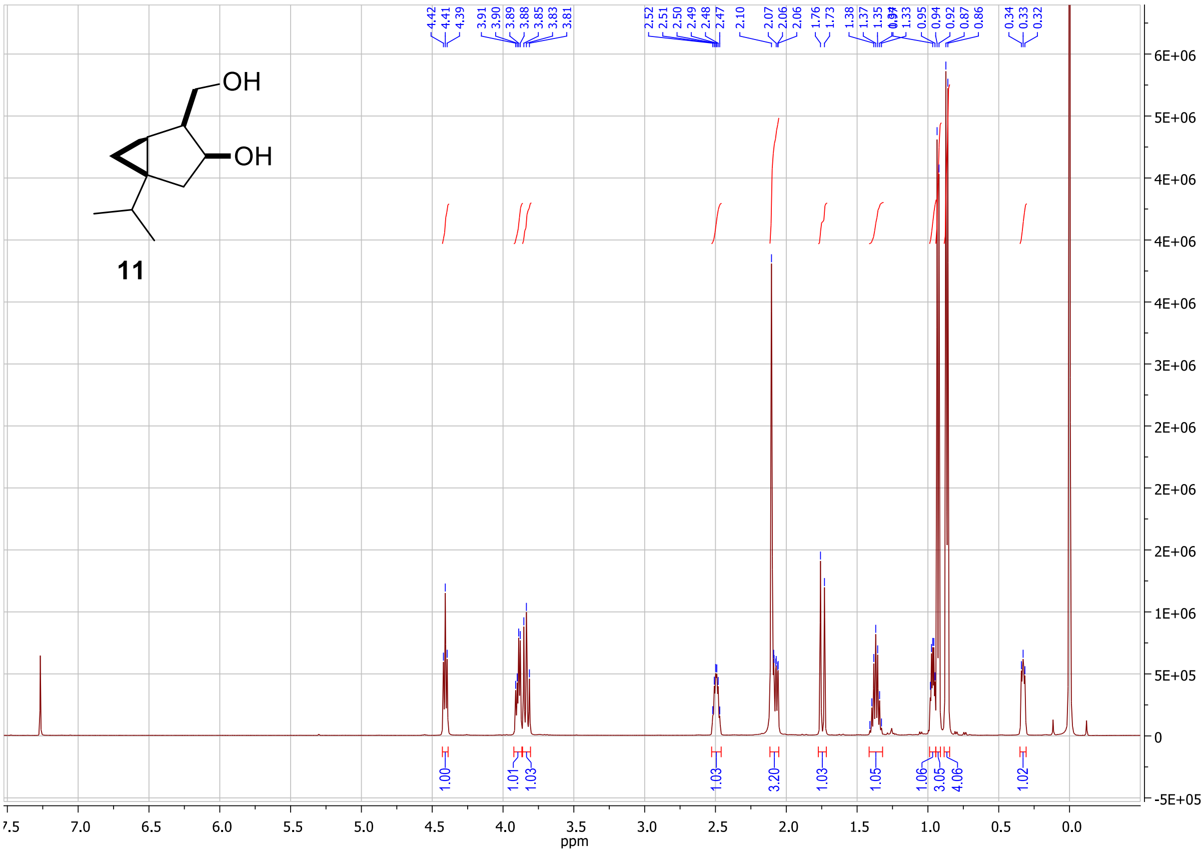

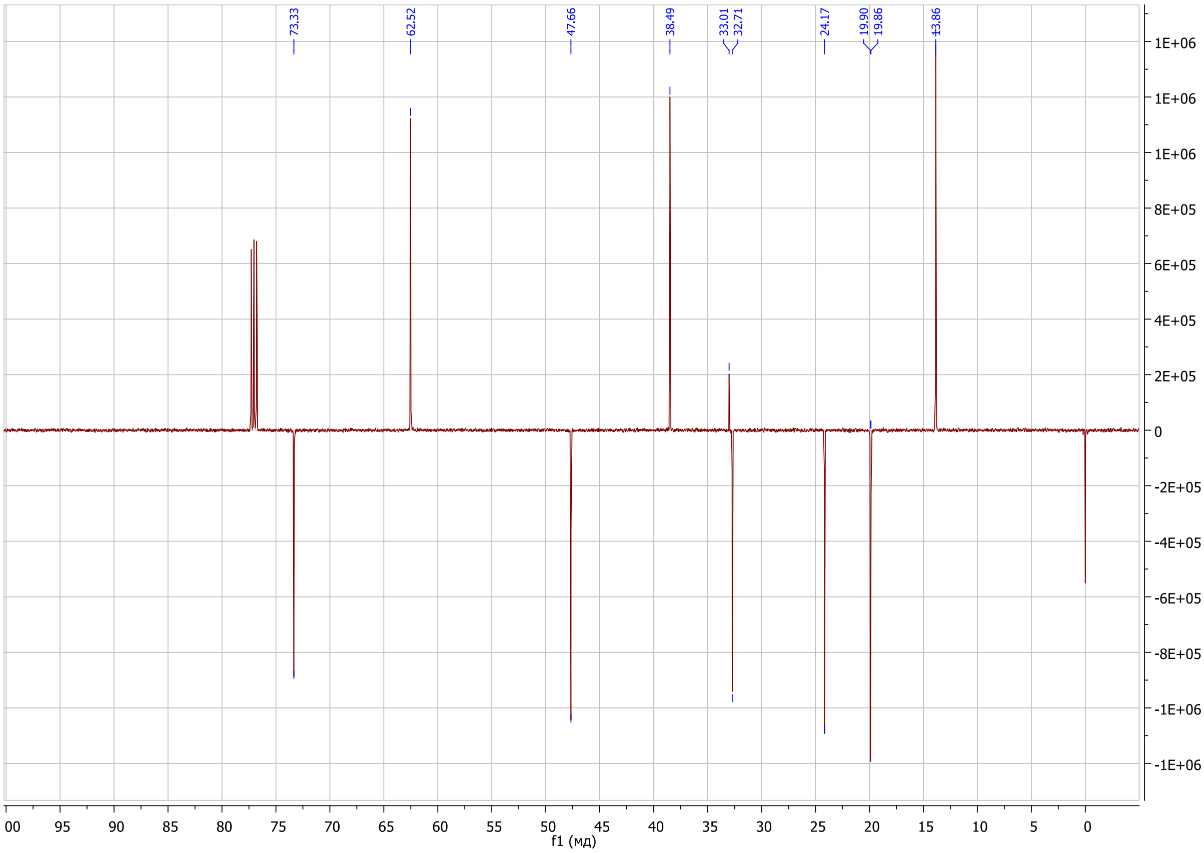

(1*R*,3*S*,5*R*)-3-Benzyloxy-1-isopropyl-4-methylenebicyclo[3.1.0]hexane (**12**)

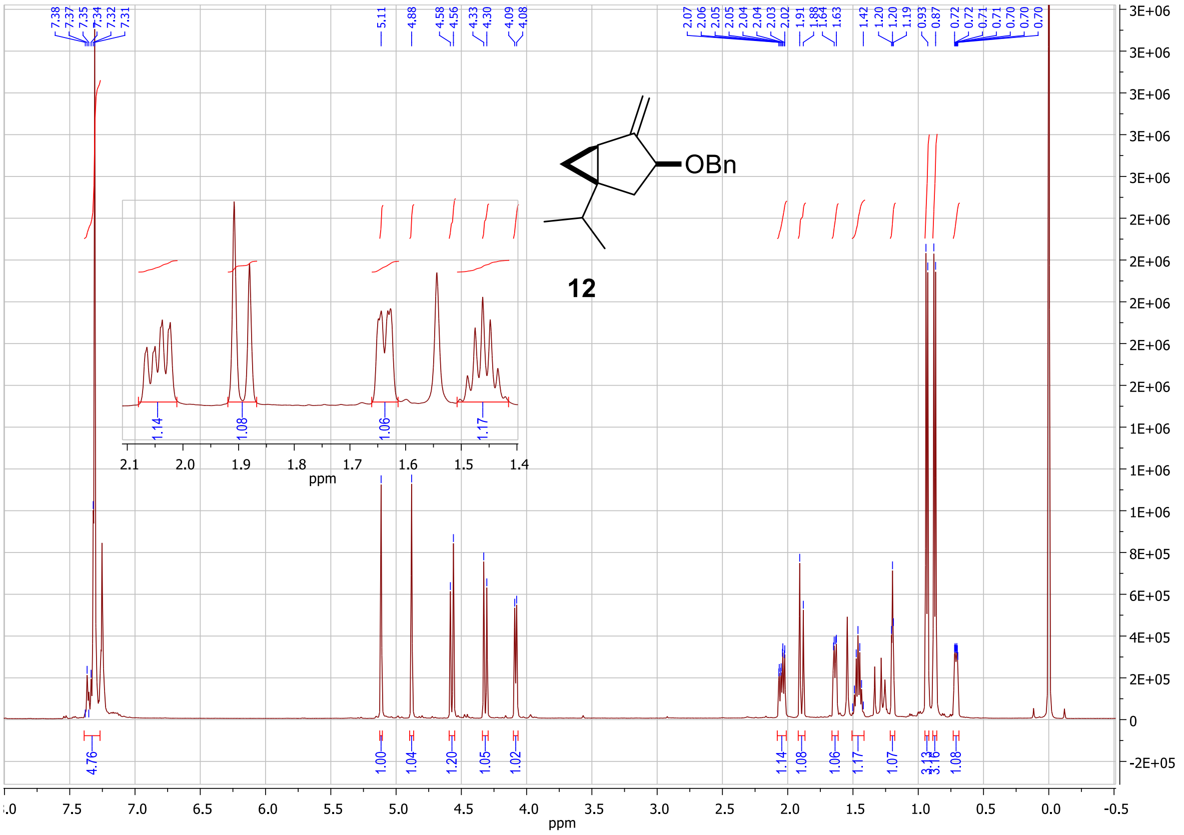

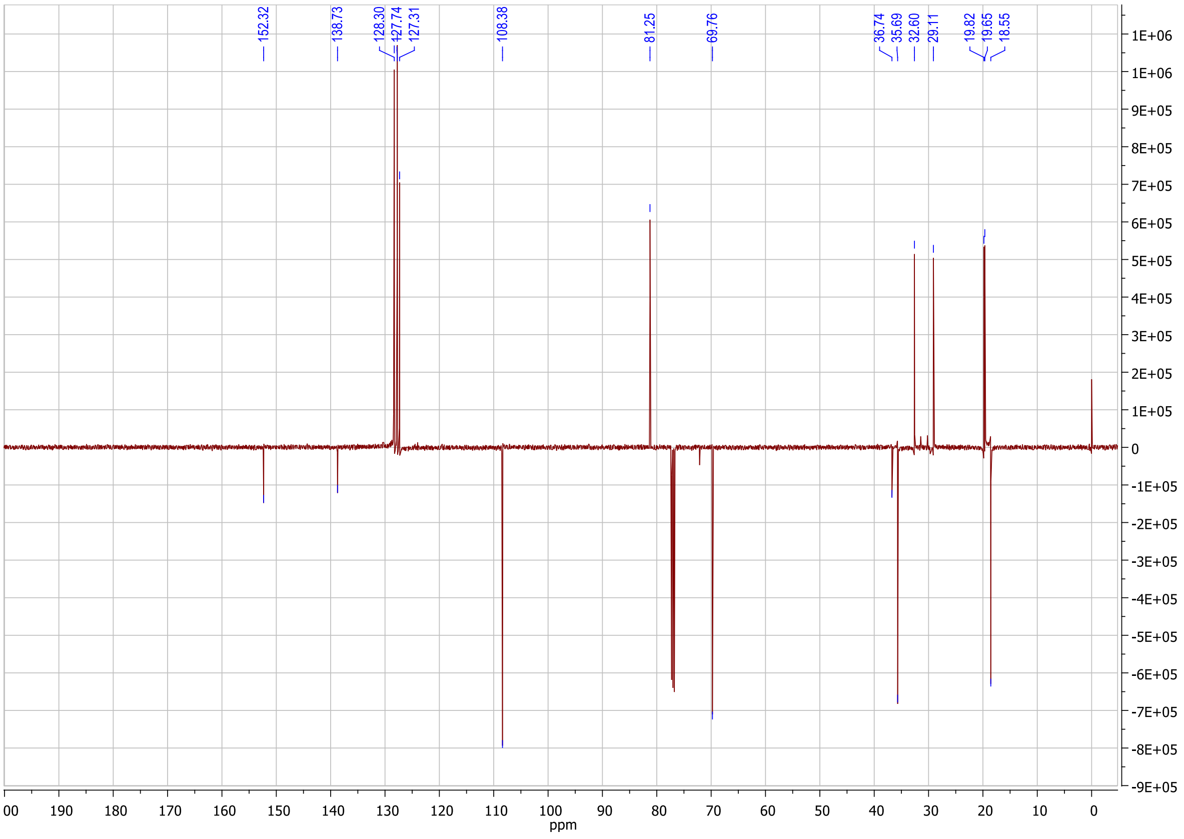

(1*R*,2*R*,3*S*,5*R*)-2-Hydroxymethyl-5-isopropylbicyclo[3.1.0]hexane-2,3-diol (**13**)

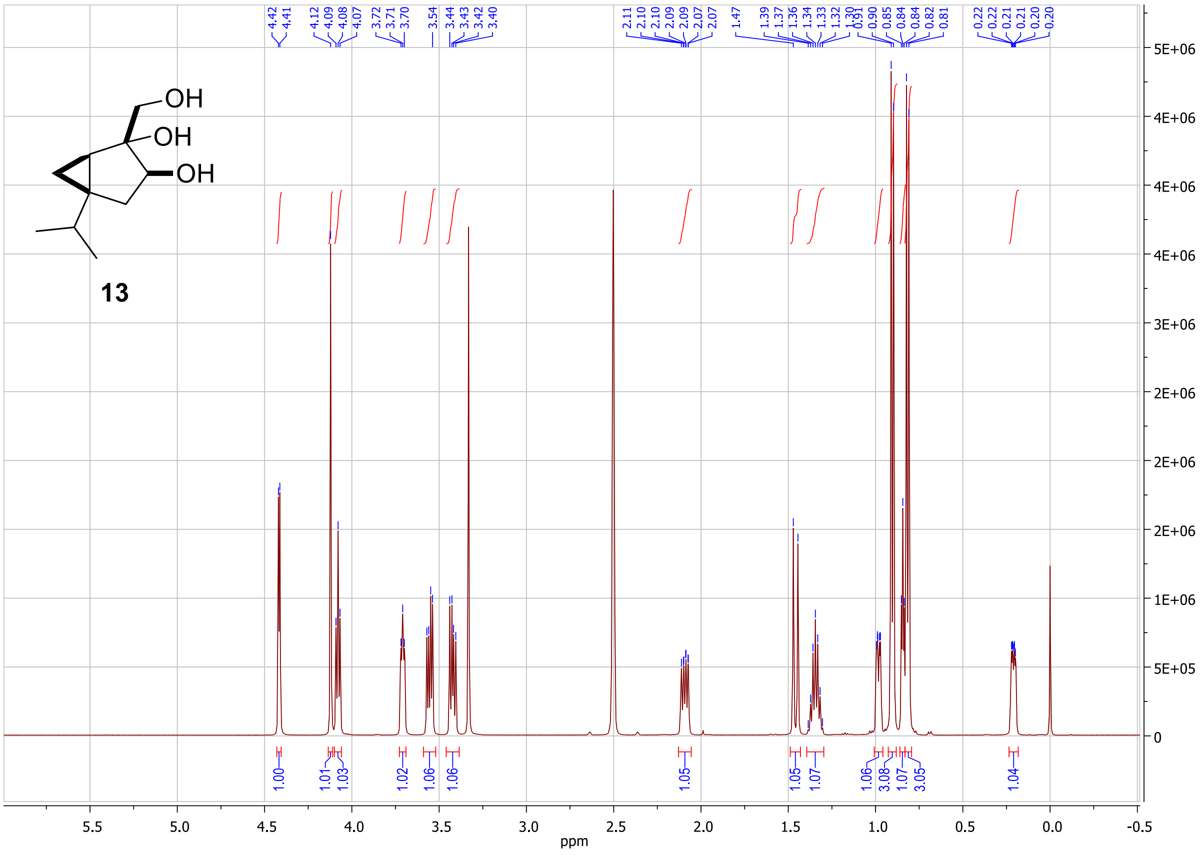

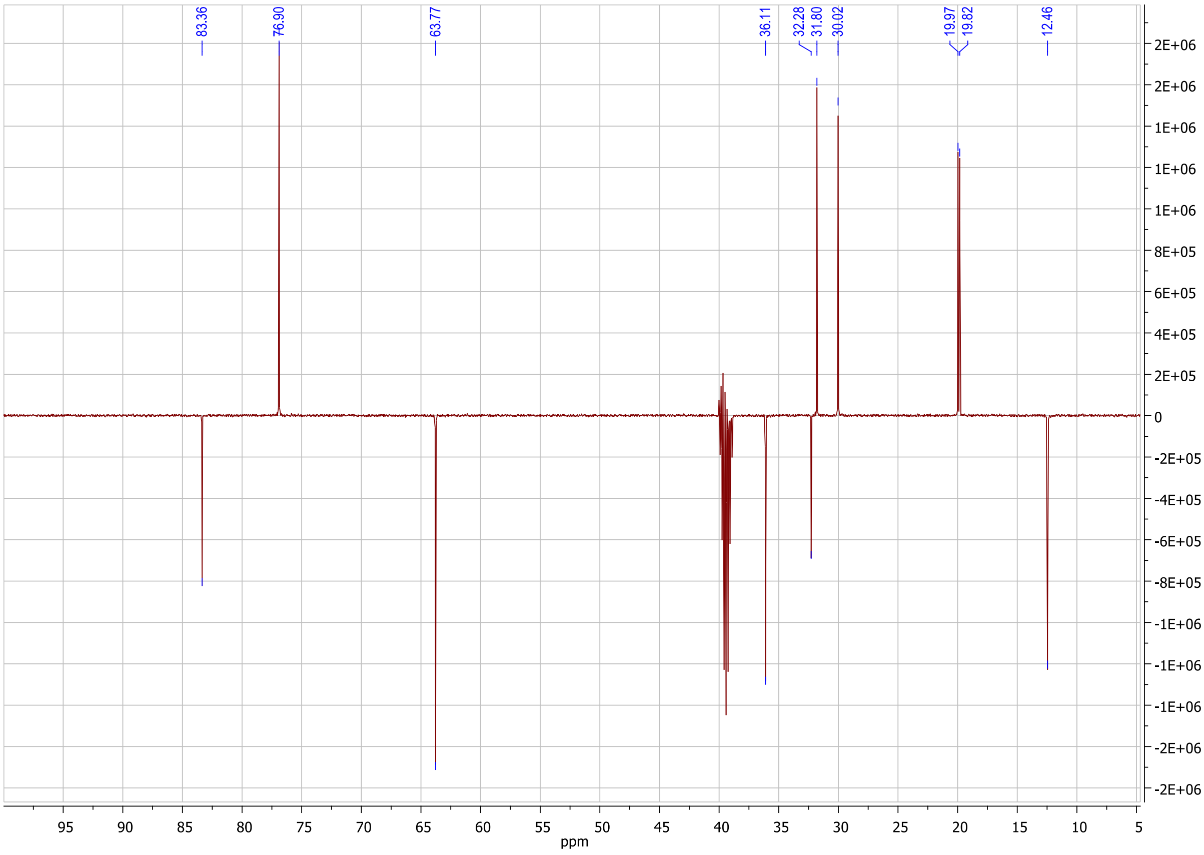

COSY spectrum of **13**

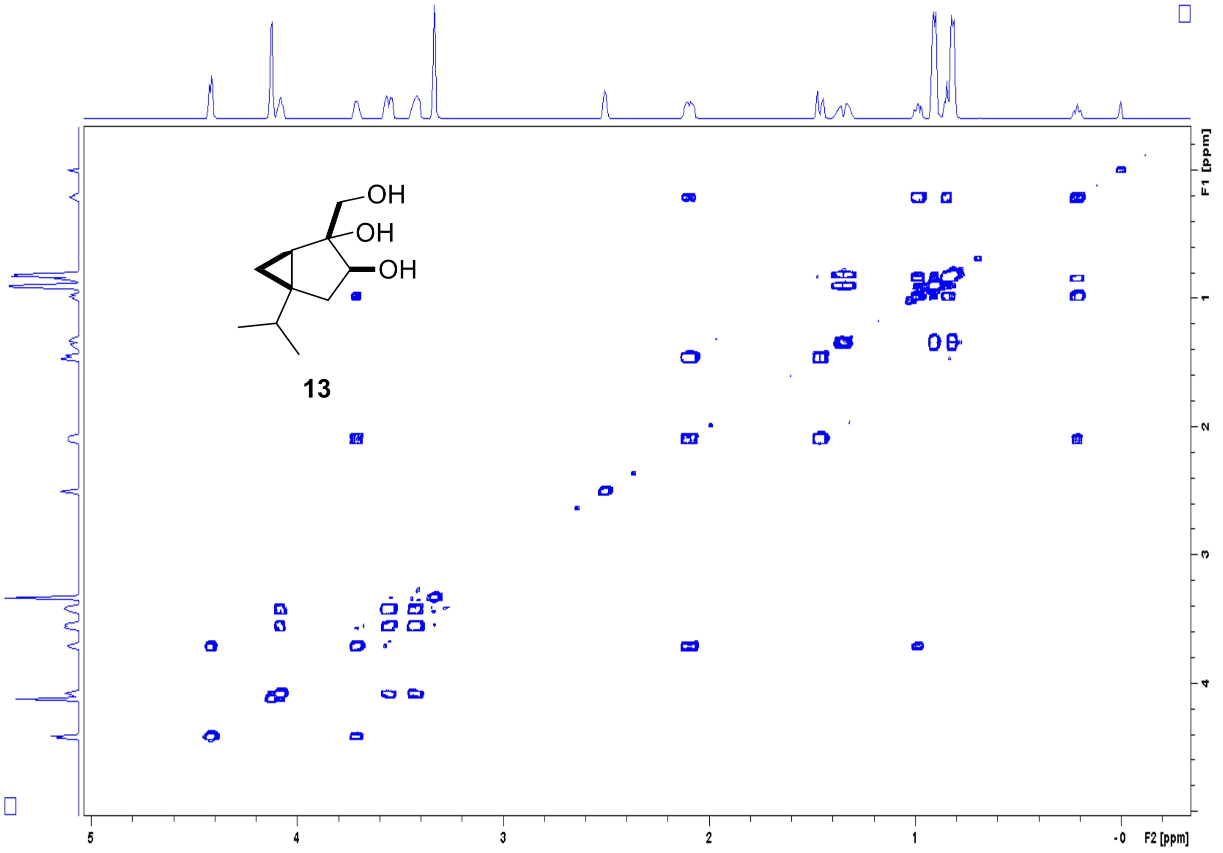

NOESY spectrum of **13**

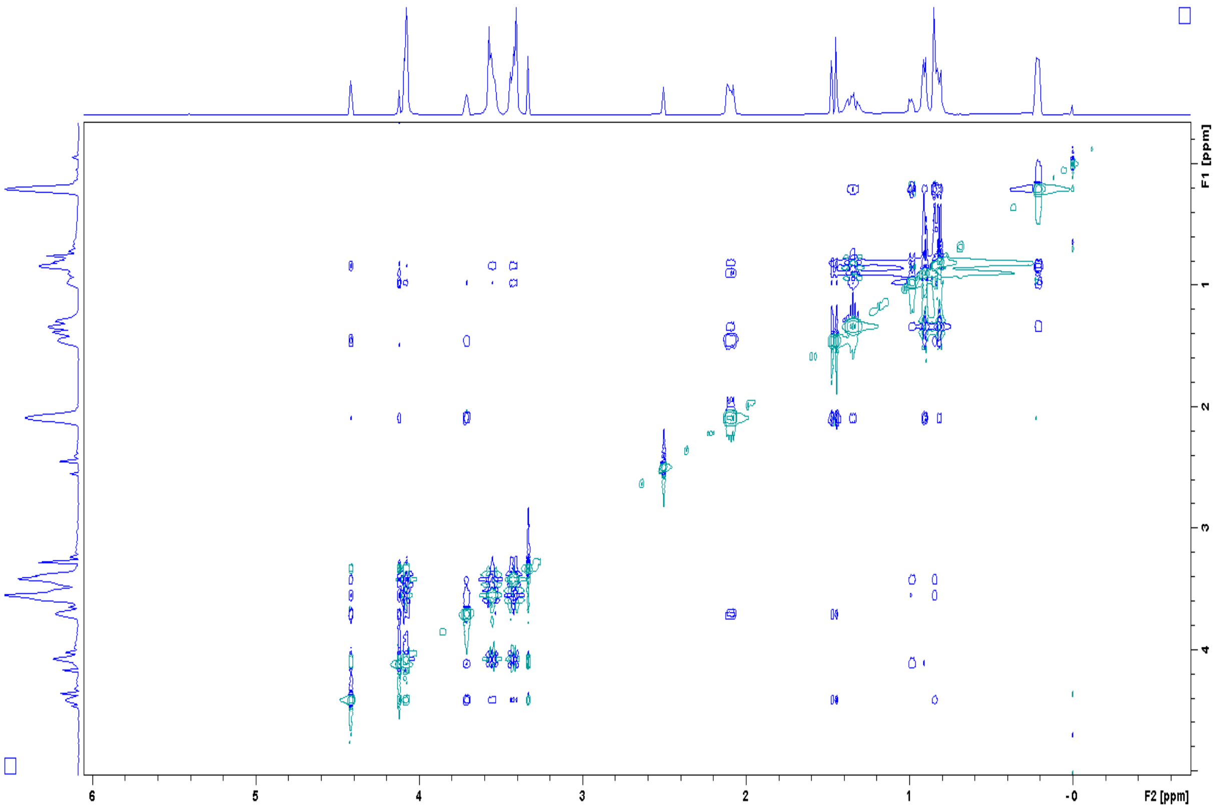

HSQC spectrum of **13**

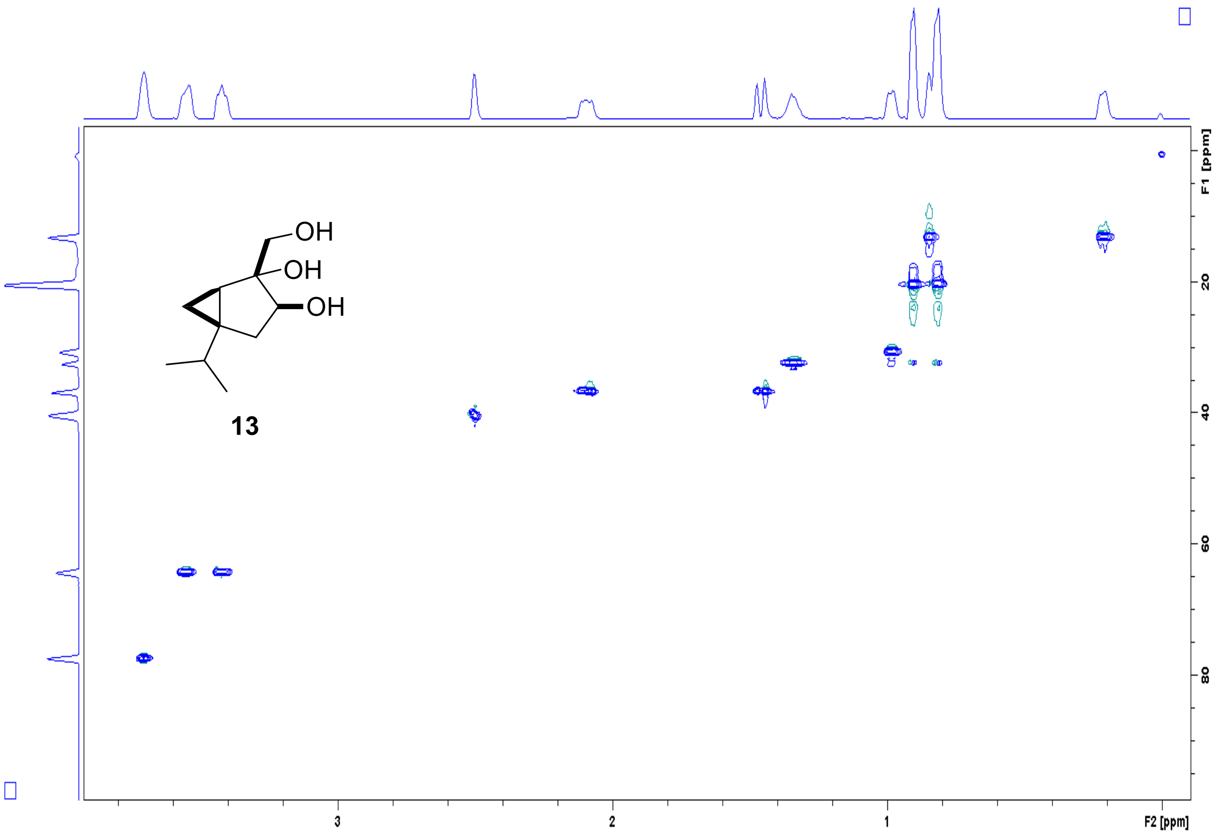

HMBC spectrum of **13**

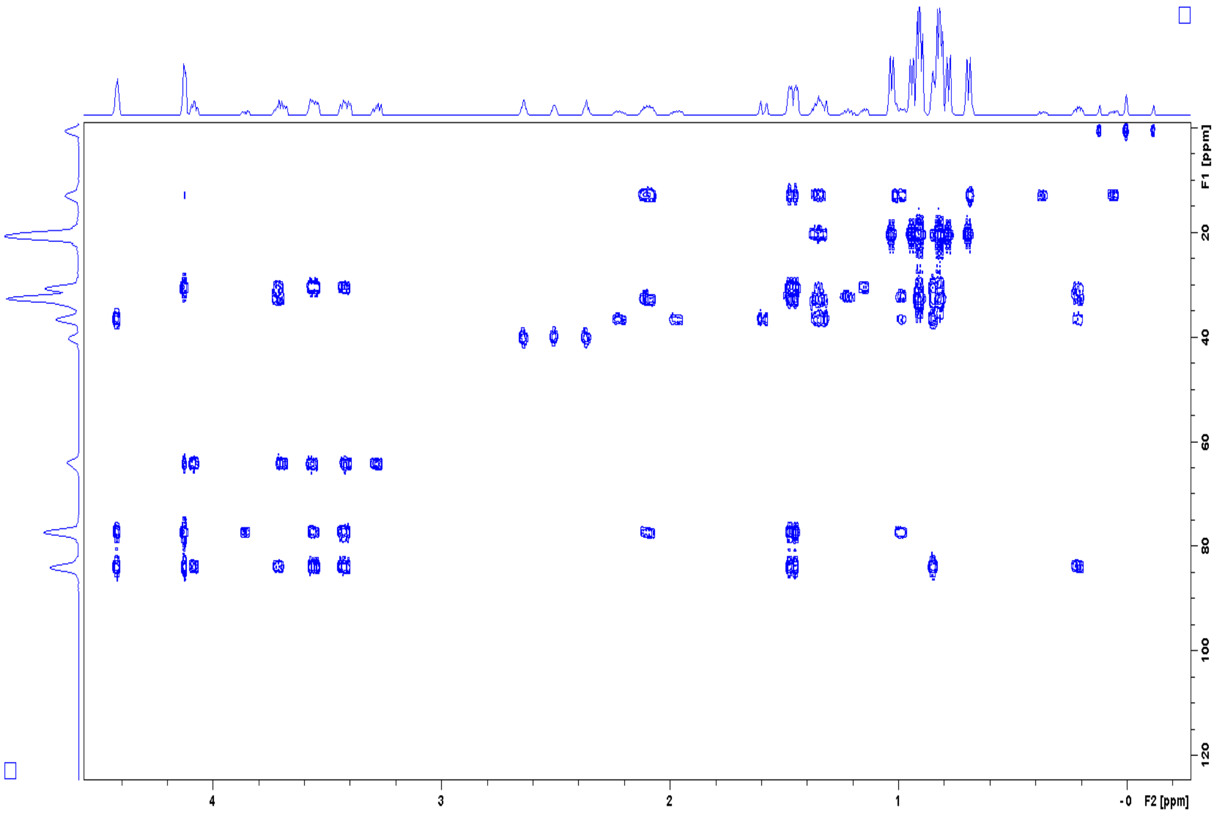

(1*R*,2*R*,3*S*,5*R*)-3-Benzyloxy-2-hydroxymethyl-5-isopropylbicyclo[3.1.0]hexan-2-ol (**14**)

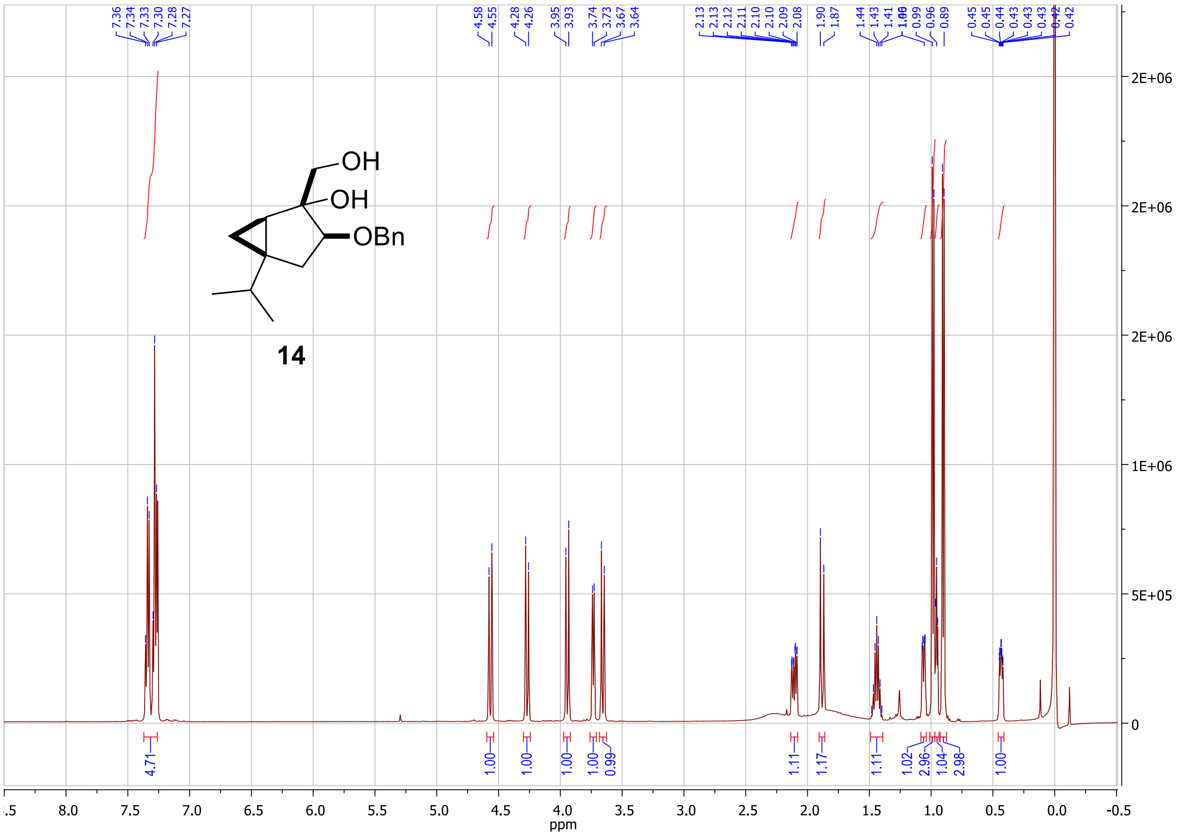

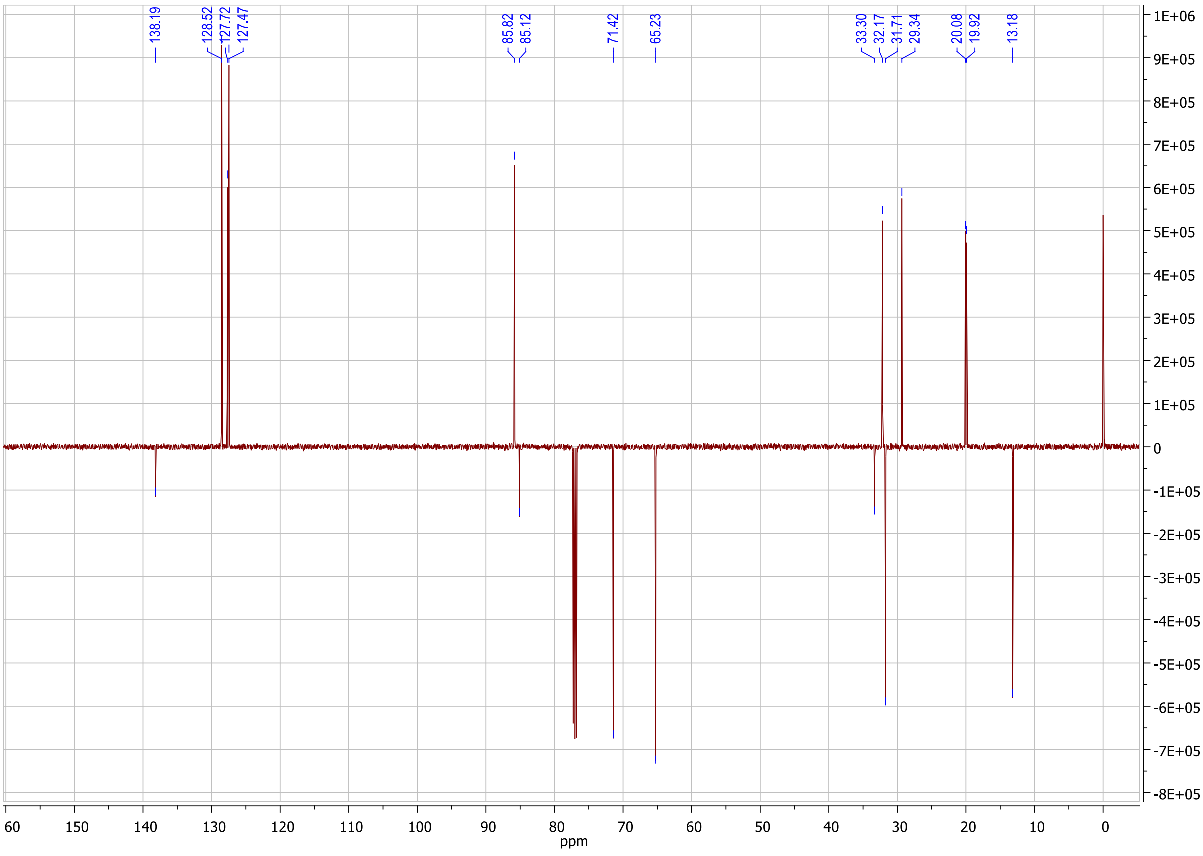

((1*R*,2*S*,3*S*,5*R*)-2,3-Dihydroxy-5-isopropylbicyclo[3.1.0]hexan-2-yl)methyl 3-chlorobenzoate (**17**)

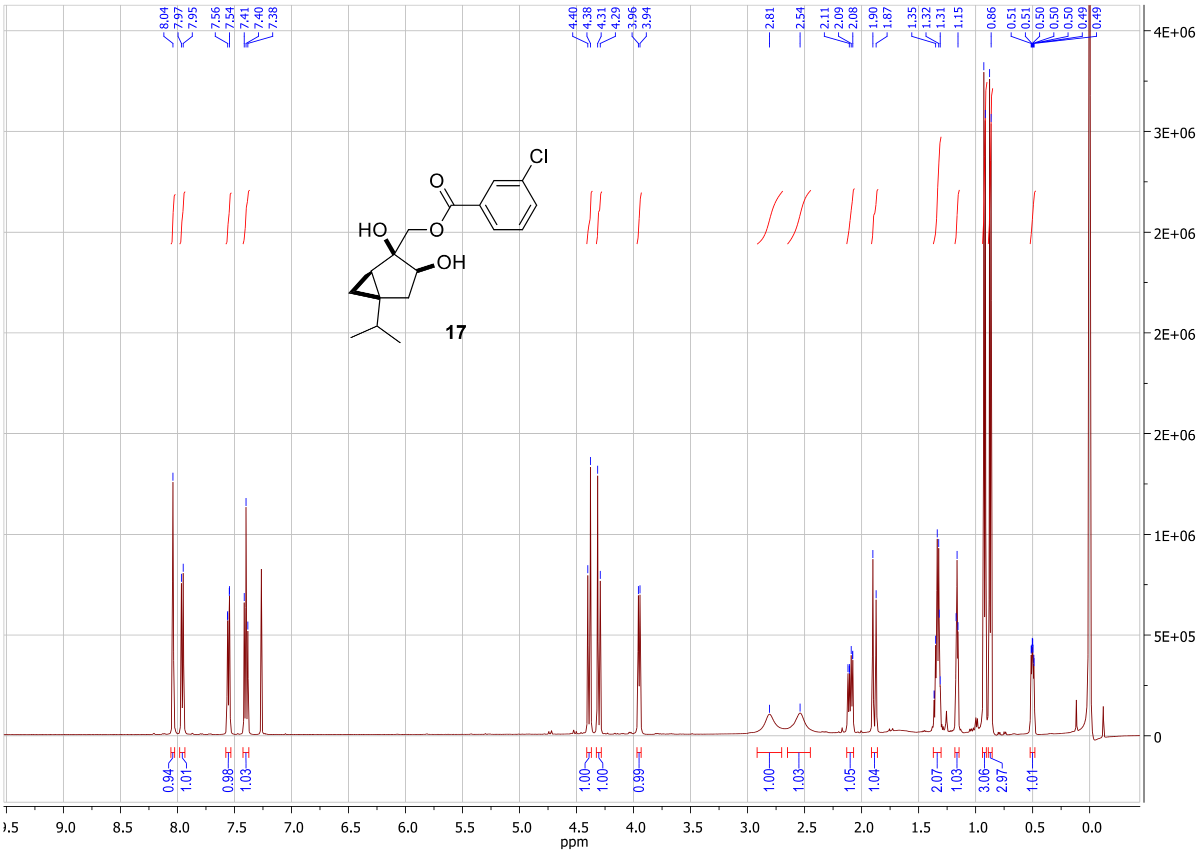

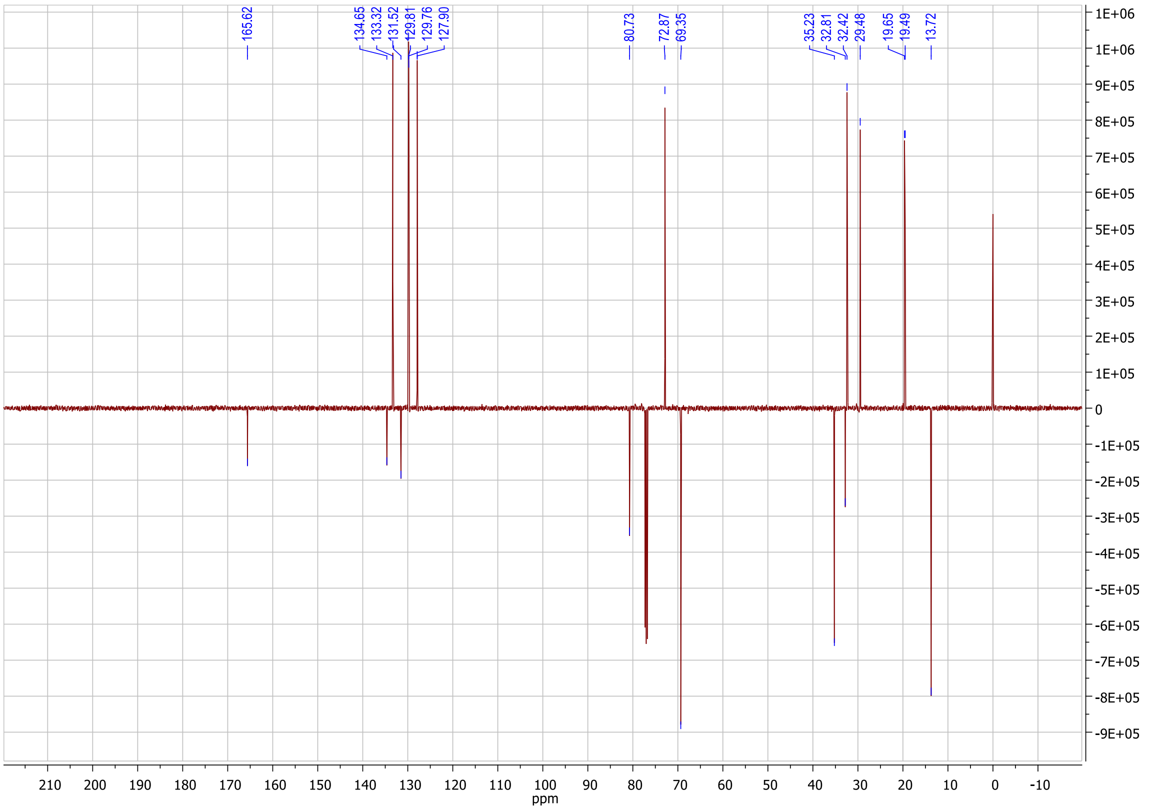

(1*R*,3*S*,4*S*,5*R*)-4-Hydroxy-4-hydroxymethyl-1-isopropylbicyclo[3.1.0]hexan-3-yl 3-chlorobenzoate (**18**)

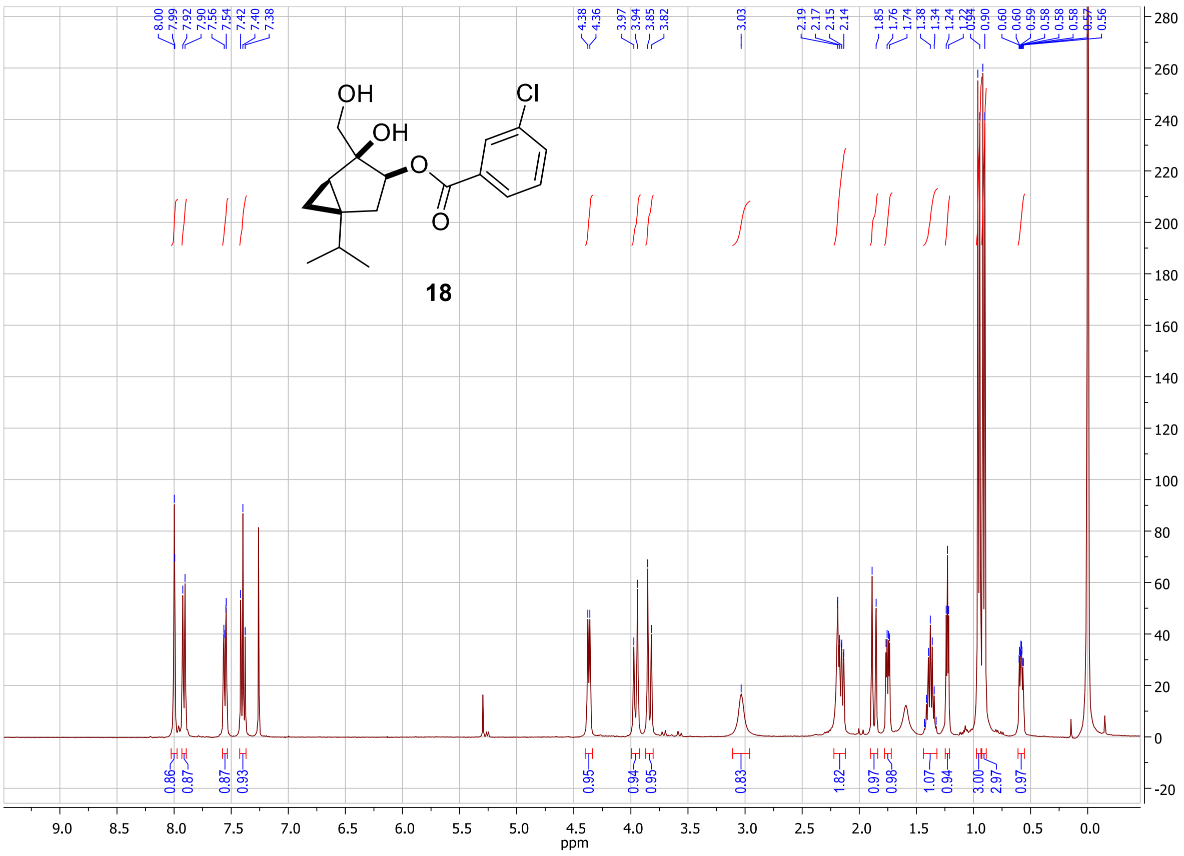

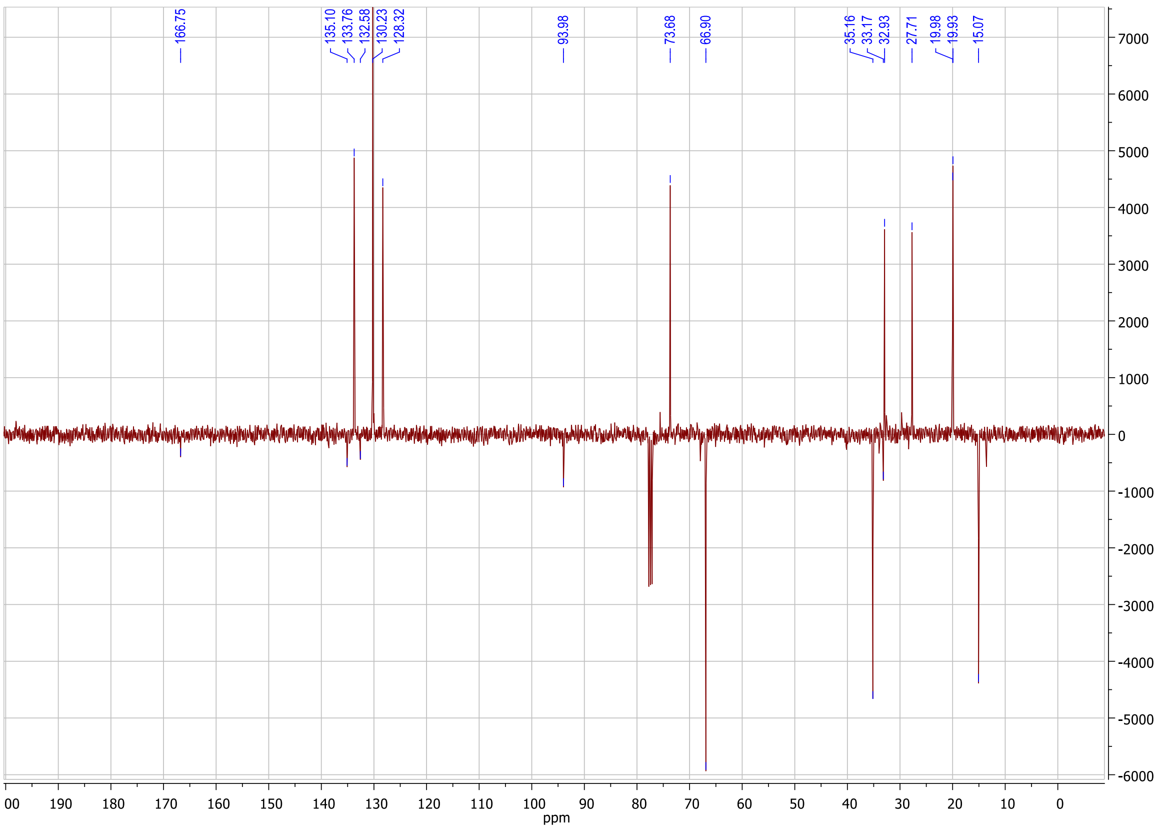

(1*R*,2*S*,3*S*,5*R*)-2-Hydroxymethyl-5-isopropylbicyclo[3.1.0]hexane-2,3-diol (**19**)

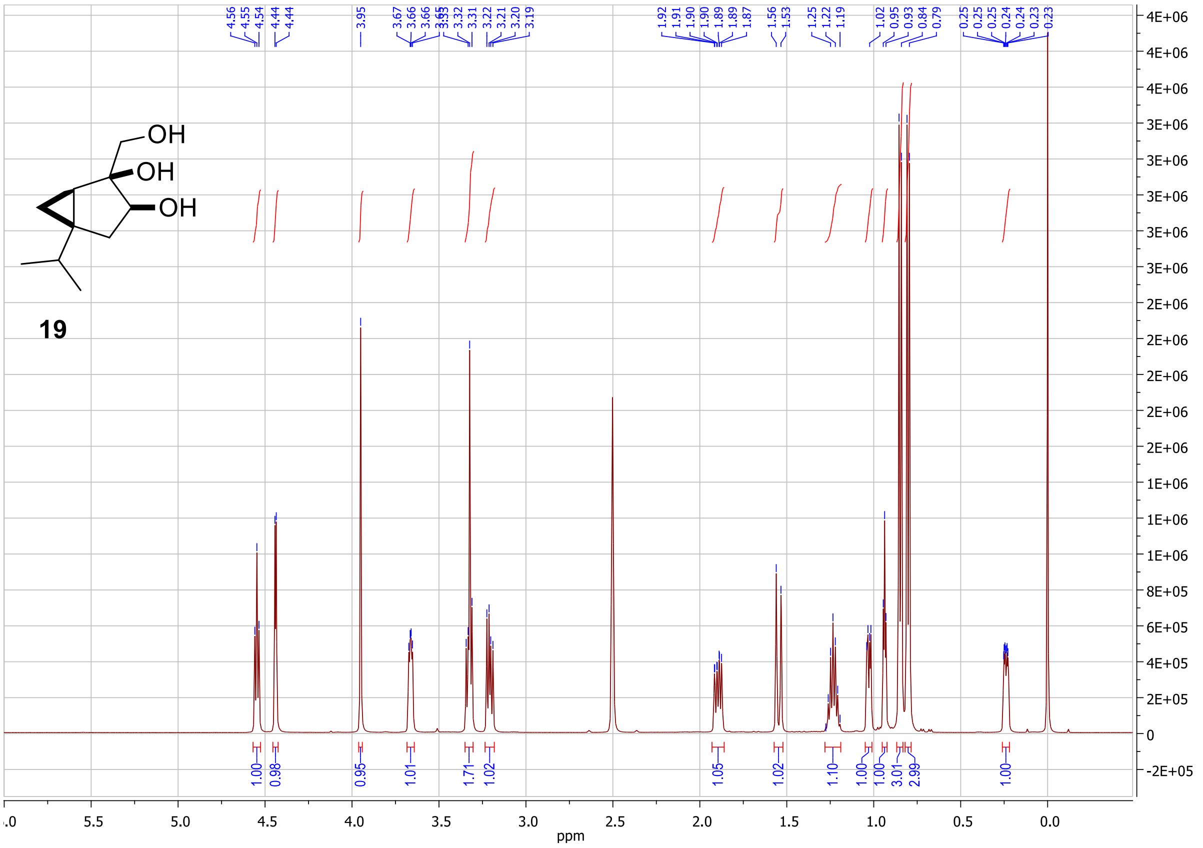

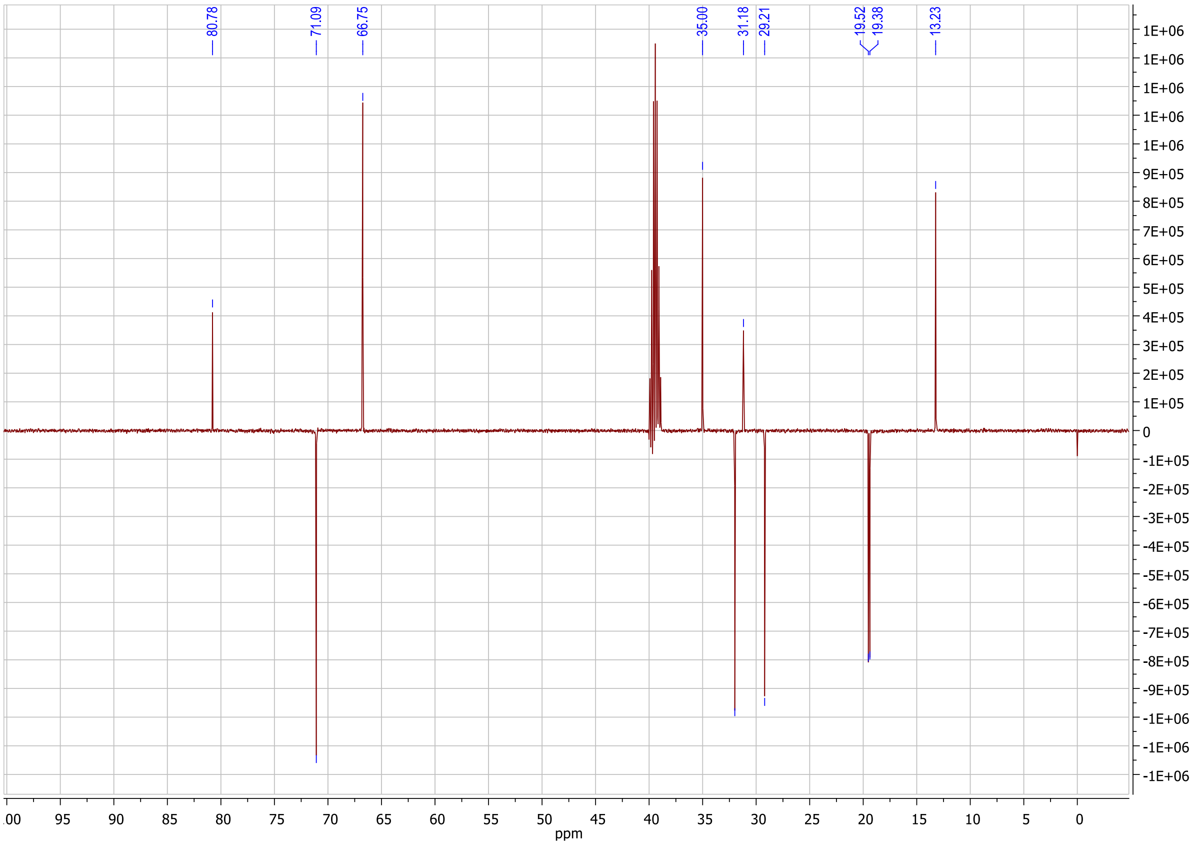

COSY spectrum of **19**

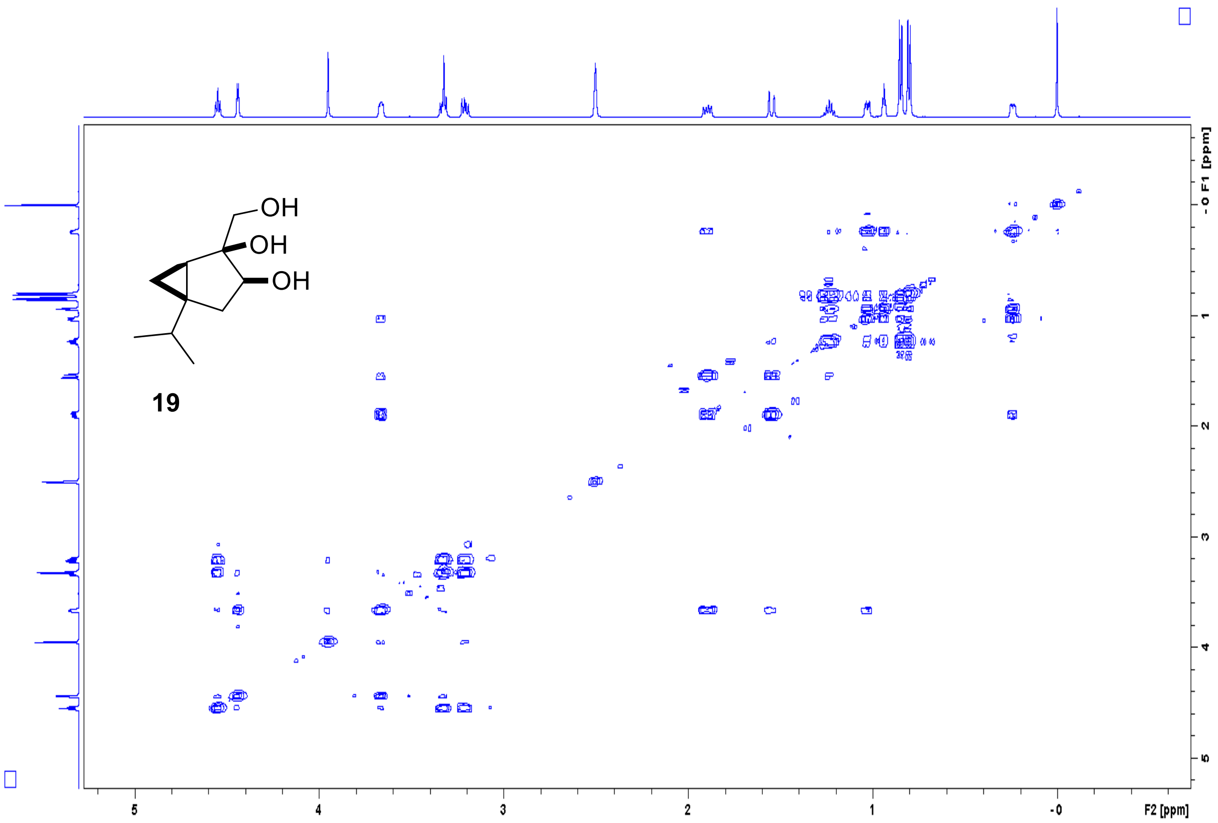

NOESY spectrum of **19**

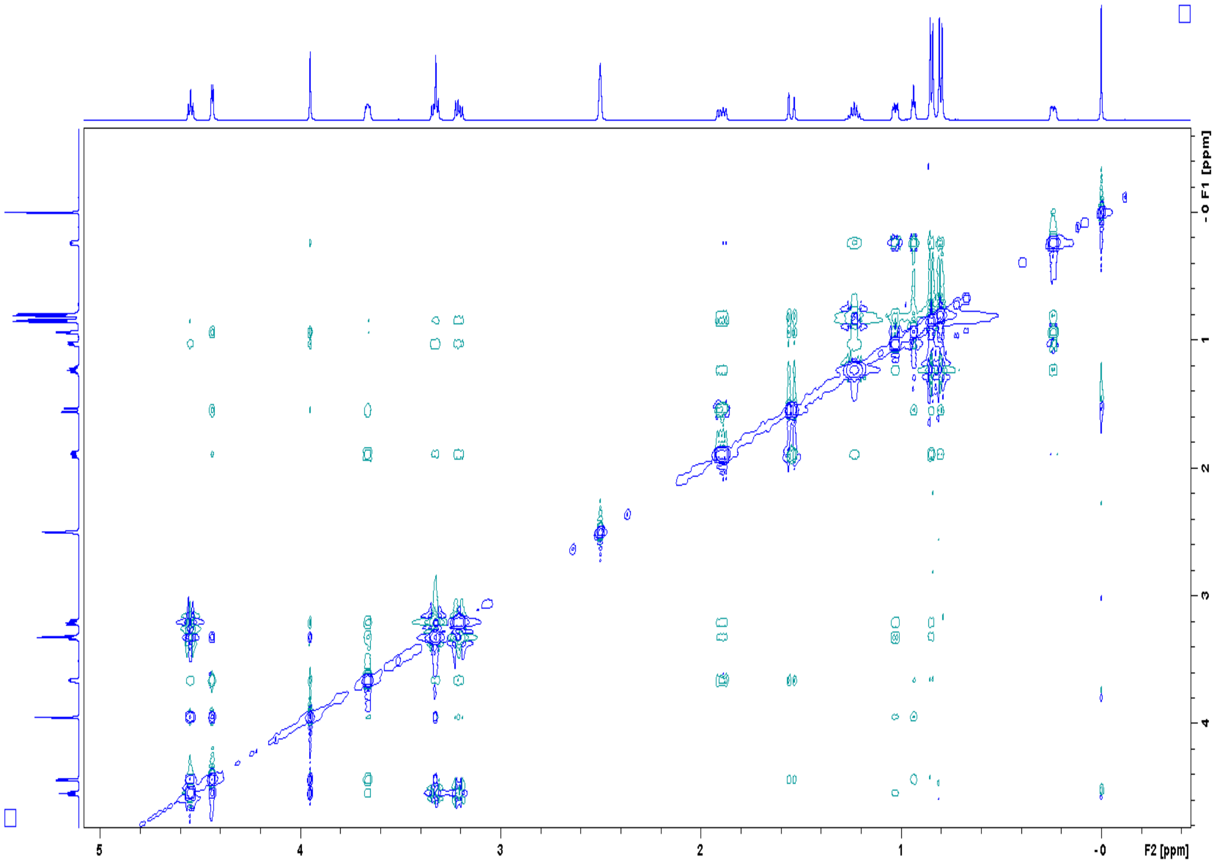

HSQC spectrum of **19**

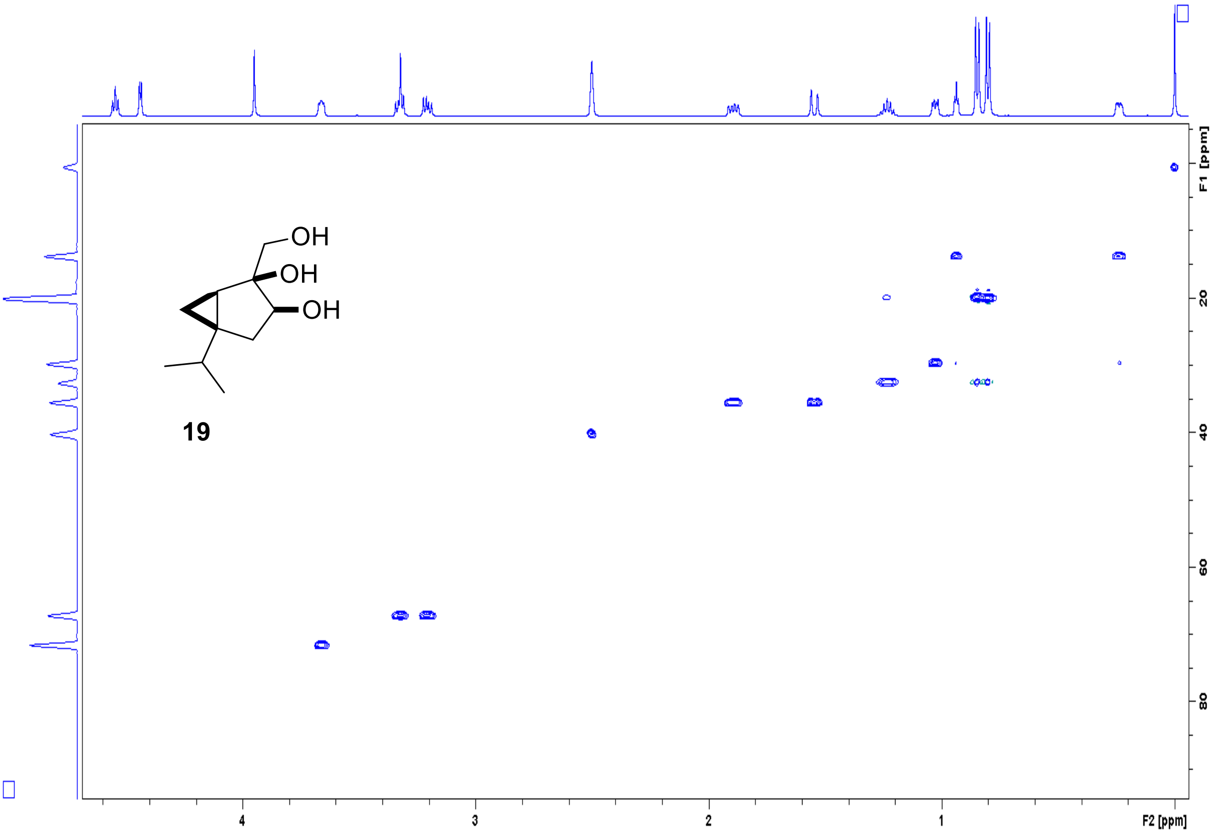

HMBC spectrum of **19**

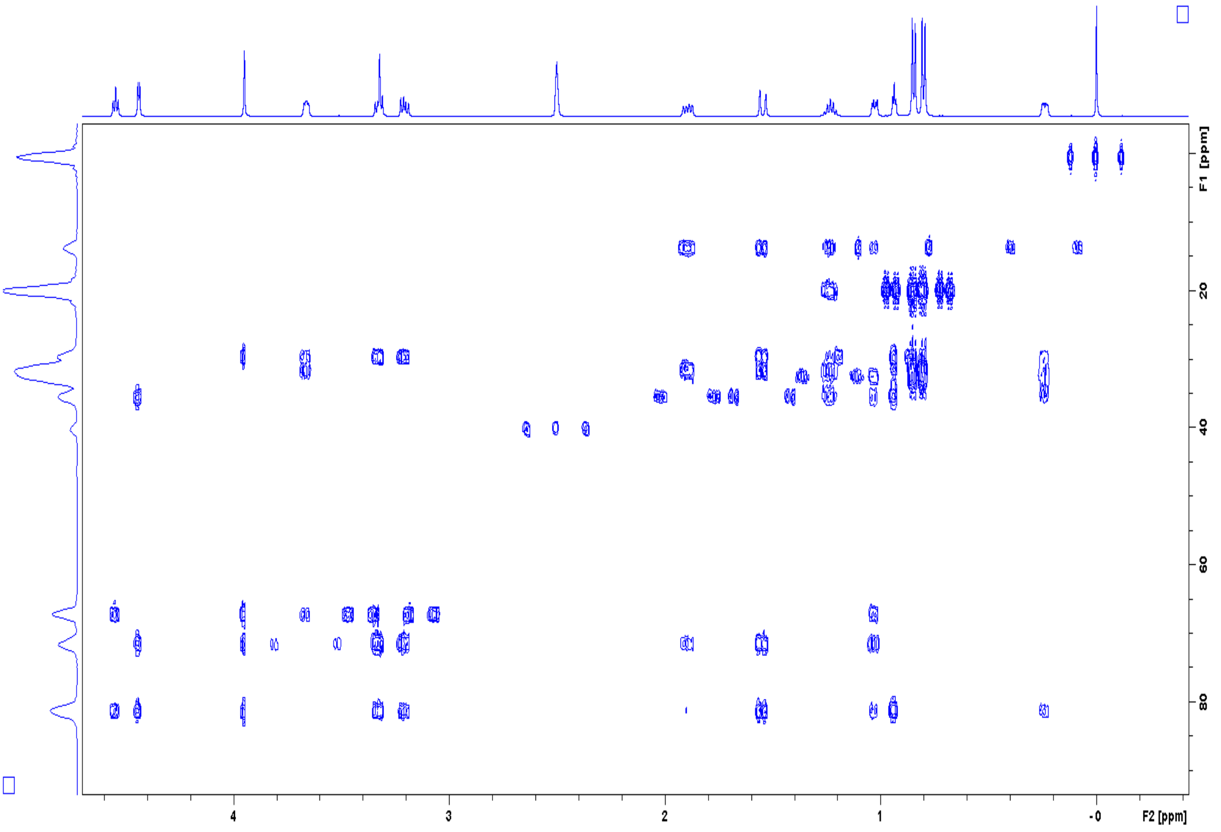

CCDC number 1814013 contains the supplementary crystallographic data for this paper. These data can be obtained free of charge via http://www.ccdc.cam.ac.uk/conts/retrieving.html (or from the CCDC, 12 Union Road, Cambridge CB2 1EZ, UK; Fax: +44-1223-336033; E-mail: deposit@ccdc.cam.ac.uk).

Figure S1. Crystal structure of diol **11**.

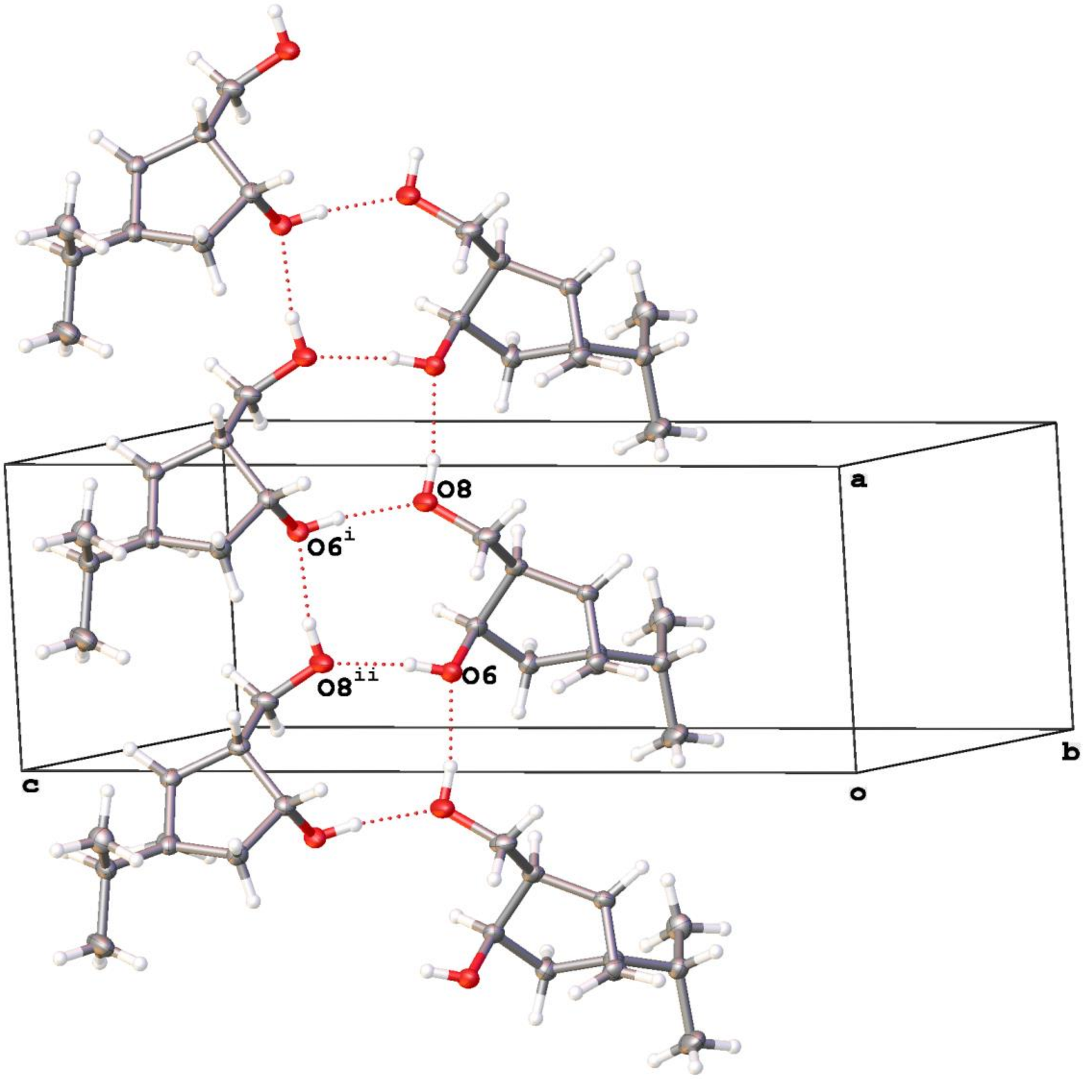

Supplement: Supplementary file 1 [file molecules-23-00771-s001.pdf]
